# Supplementary material for: Streptococcus pyogenes EVs induce the alternative inflammasome via caspase-4/-5 in human monocytes
Source: EMBO Rep. 2025 Sep 8;26(19):4847–85. doi: 10.1038/s44319-025-00558-7 (PMC12508482; doi:10.1038/s44319-025-00558-7)
Supplement: Supplementary file 1 — Appendix [file 44319_2025_558_MOESM1_ESM.pdf]

Appendix

Streptococcus pyogenes EVs induce the alternative inflammasome via Caspase-4/-5 in human monocytes

Table of Content

- Appendix Figure S1 p. 2
- Appendix Figure S2 pp. 3-4
- Appendix Figure S3 p. 5

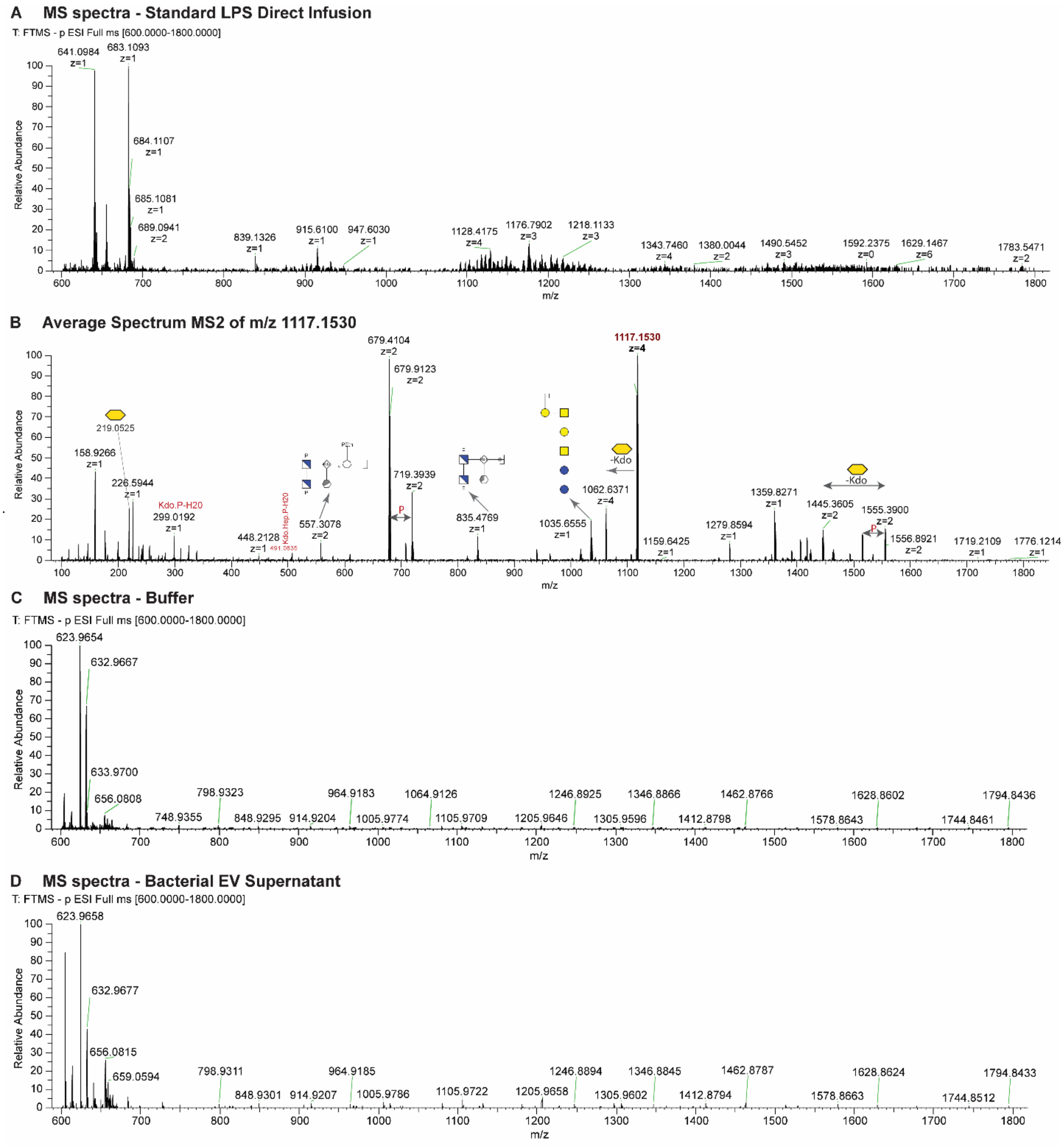

Mass to charge	Ion (proposed composition)	Type
143.0360414	Z-Hex1-B	BZ
219.0075776	KDO1-B	B
708.4134473	ZY-HexN1KDO2P1	ZY
835.4769324	^{3,5}XY-HexN2KDO1P2	^{3,5}X_{KDO}Y
939.2908211	^{2,5}XZ-HexN2KDO1P2	^{2,5}X_{KDO}Z

Appendix Figure S1. Direct Infusion ESI HCD MS/MS analysis. (A) MS-full scan from *E. coli* 055:B5 LPS in positive ionization mode. **(B)** MS/MS scan of precursor m/z 117.1530. The low m/z region of the HCD MS/MS spectra corresponds to the LPS bearing Kdo (m/z 219.05) and phosphates (m/z 290.01). Other ions corresponding to 055:B5 LPS structure are indicated in the MS/MS spectra with proposed composition. Additional annotations are provided in the table below. **(C)** MS-full scan of buffer used for the isolation of bacterial EVs and **(D)** bacterial EV supernatant.

A

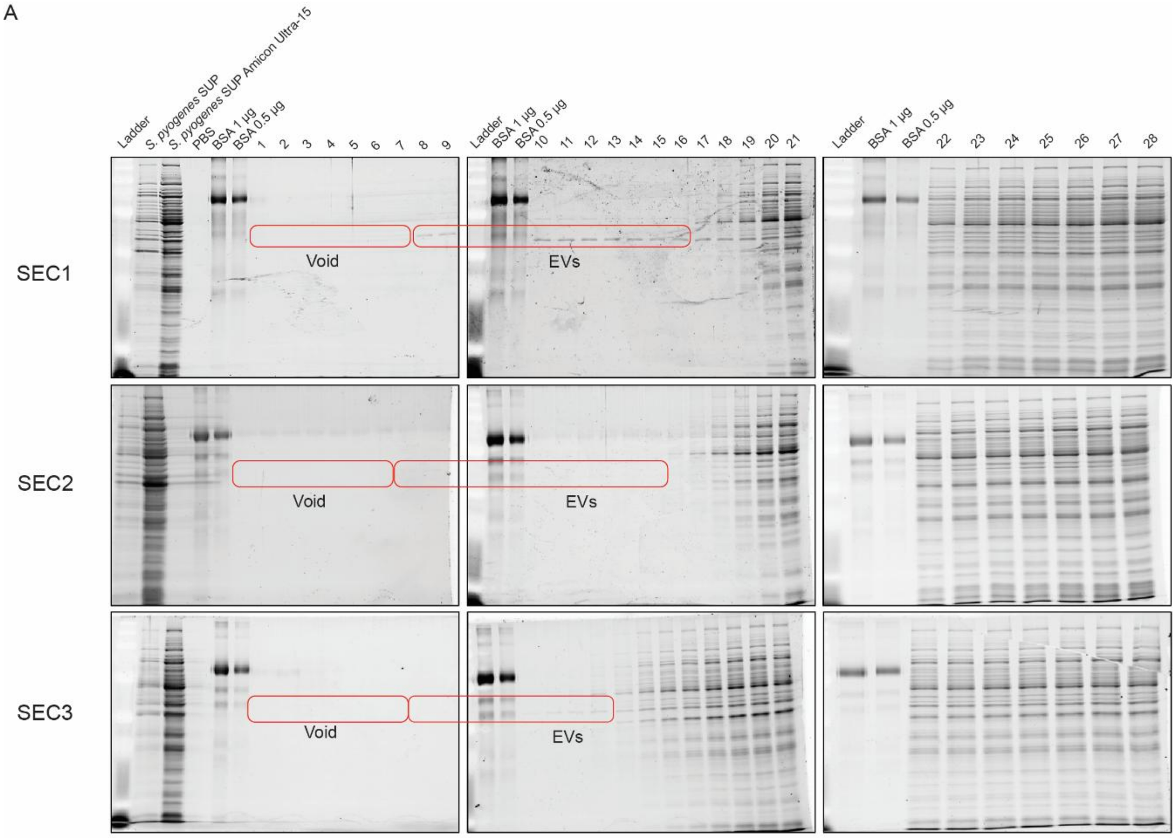

B

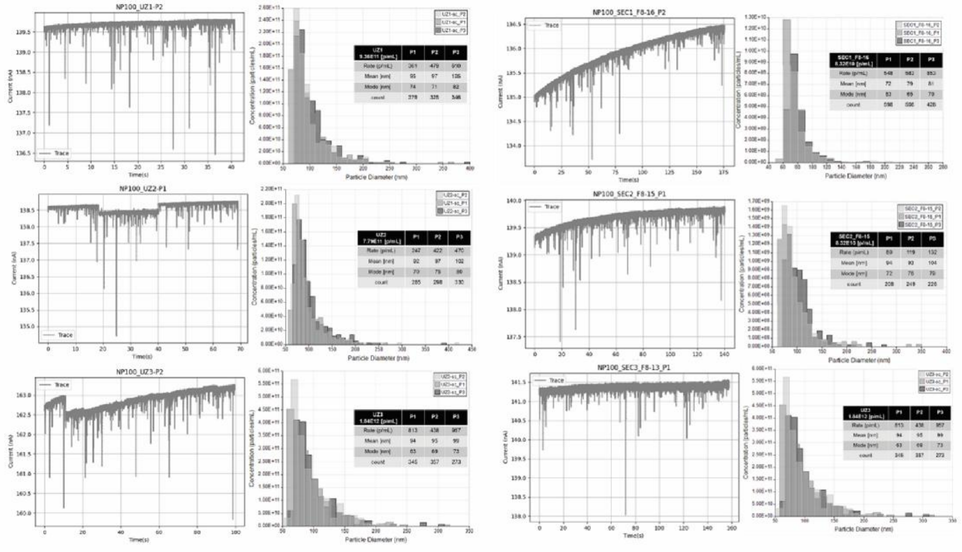

C

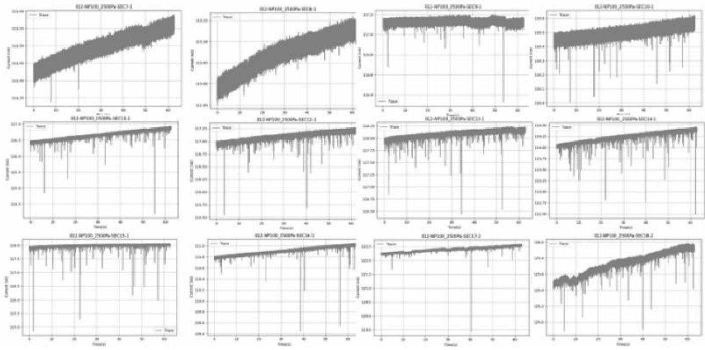

SEC_indivdual fractions	Rate (p/mL)	Mean [nm]	Mode [nm]	count	Conc [p/mL]
F7	1.9	NaN	NaN	2	NaN
F8	1.9	NaN	NaN	2	NaN
F9	56	78	71	56	5.3E09
F10	75	79	70	79	7.5E09
F11	61	79	63	64	6.1E09
F12	242	85	67	253	2.4E10
F13	72	88	103	103	3.2E10
F14	383	85	68	402	1.1E11
F15	339	85	67	354	1.0E11
F16	62	87	72	65	1.8E10
F17	173	89	71	182	5.1E10
F18	282	93	82	296	3.4E9

Appendix Figure S2. *Spy* EV isolation using Size exclusion chromatography (SEC). (A) SYPRO Ruby stained polyacrylamide gels (12%) of 28 SEC fractions eluted from ultrafiltrated *S. pyogenes* culture supernatants (SUP, Amicon Ultra-15 concentrate). **(B)** UC- and pooled SEC-EV-quantification with TPRS/Exoid. Plots of representative blockage events over time, termed traces with size & concentration histograms and quantification results inscribed in respective tables (UC-EVs on the left, pooled SEC-EVs on the right). Note that with the Exoid, traces are acquired at 3 different pressures (P1,2,3). P1 is 200Pa lower than P2. P3 is 300Pa higher than P2 in one duty cycle. Appropriately diluted samples were measured with a NP100 and 47mm stretch and CPC100 calibration particles. **(C)** Characterization of Individual SEC-fractions. Trace-plots of individual SEC-fractions at one pressure (2500Pa, 1200mV, 60sec on NP100) with available quantification results summarized in a table.

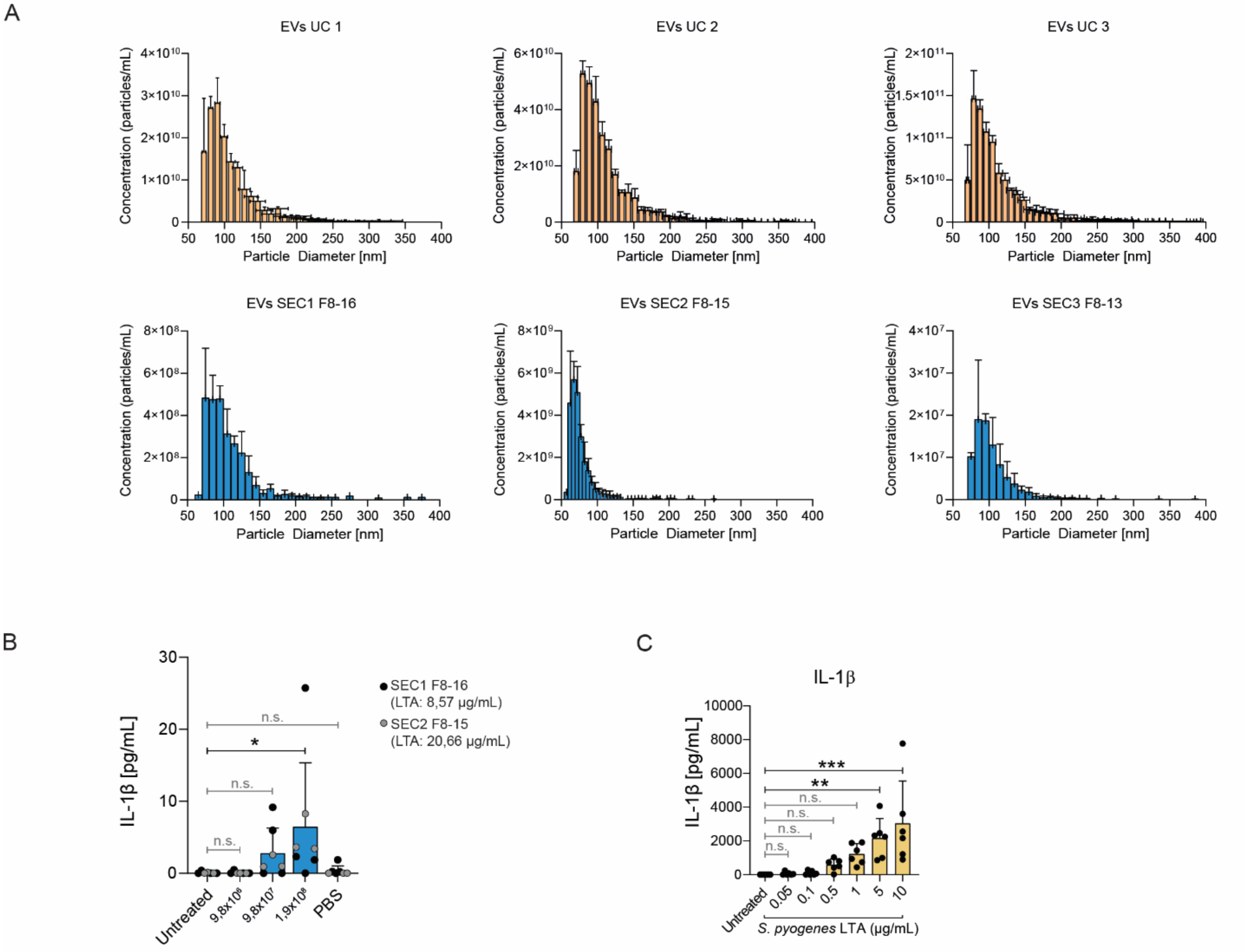

Appendix Figure S3. SEC EV quantification and IL-1β response (A) Size and concentration of particles present in EV UC as well as SEC EV batches. Bars represent the mean ±SD of three dilutions measured. **(B)** IL-1β released by human monocytes in response to *Spy* EVs isolated using SEC. Bars represent the mean ±SD of seven biological replicates with pooled EV fractions from 2 independent SECs (black circles: SEC1 F8-16; grey circles: SEC2 F8-15). *P=0.0354.* **(C)** IL-1β released by human monocytes stimulated with increasing amounts of *Spy* LTA for 18 h. Bars represent the mean ±SD of six biological replicates. *P=0.0073* (5 µg/mL)*, P=0.0002* (10 µg/mL). **Data information:** (BC) One-way ANOVA was applied with Holm-Šídák correction for multiple comparisons. *p≤0.05, **p≤0.01, ***p≤0.001, $^{n.s.}$ not significant
