# Supplementary material for: Streptococcus pyogenes EVs induce the alternative inflammasome via caspase-4/-5 in human monocytes
Source: EMBO Rep. 2025 Sep 8;26(19):4847–85. doi: 10.1038/s44319-025-00558-7 (PMC12508482; doi:10.1038/s44319-025-00558-7)
Supplement: Supplementary file 5 — Source data Fig. 2 [file 44319_2025_558_MOESM5_ESM.zip › Figure_2/Figure_2E/PgRuler_Plus_Prestain_Protein_Ladder.pdf]

thermo scientific

PRODUCT INFORMATION

Thermo Scientific PageRuler Plus Prestained Protein Ladder

Pub. No. MAN0011773

Rev. Date 04 July 2019 (Rev. C.01)

Components	#26619	#26620	#26621
PageRuler Plus Prestained Protein Ladder	2 x 250 µL	10 x 250 µL	25 µL

Store at -20 °C

www.thermofisher.com

www.thermoscientific.com/pierce

For Research Use Only. Not for use in diagnostic procedures.

Introduction

The Thermo Scientific™ PageRuler™ Plus Prestained Protein Ladders are a mixture of nine recombinant proteins ranging from 10 kDa to 250 kDa. Two orange reference bands at ~70 kDa and 25 kDa and one green reference band at 10 kDa highlight the blue-stained protein ladder. The protein ladder is conveniently packaged and ready to use with no heating, diluting or additional reducing agent necessary. Lot-to-lot variation of the apparent molecular weight of prestained proteins is ~5 %.

Storage Buffer: 62.5 mM Tris•H_3PO_4 (pH 7.5 at 25 °C), 1 mM EDTA, 2 % (w/v) SDS, 10 mM DTT, 1 mM NaN_3, 33 % (v/v) glycerol.

Important Product Information

- Do not boil the protein ladder.
- The protein ladder can be stored at 4ºC for up to 3 months.
- For precise protein MW determination use the PageRuler Broad Range Unstained Protein Ladder (#26630).

Migration Patterns of PageRuler Plus Prestained Protein Ladder

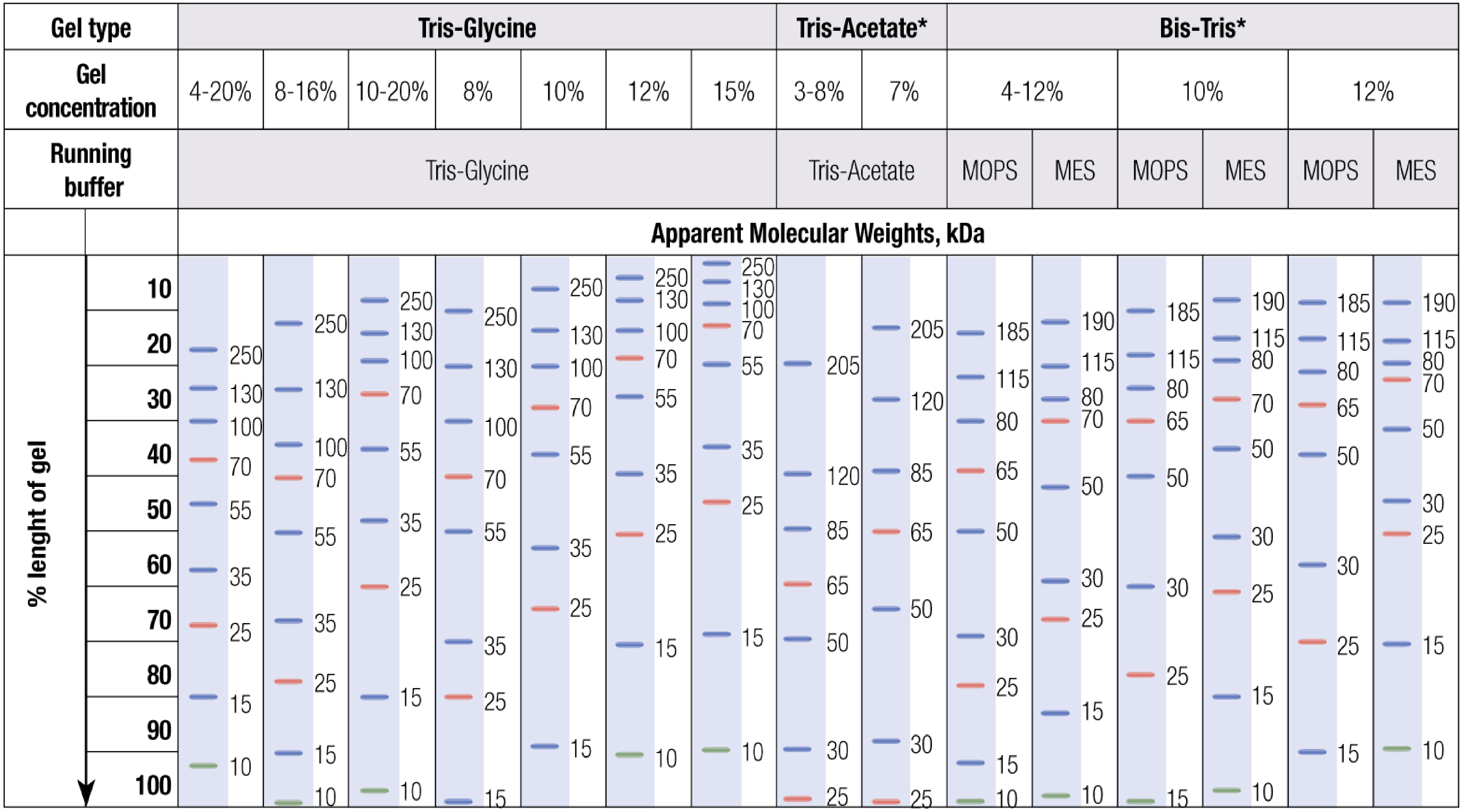

* migration patterns were determined using respective NuPAGE® precast gels.

Recommendations for Loading

1. Thaw the ladder either at room temperature or at 37-40 °C for a few minutes to dissolve precipitated solids.
   Do not boil!
2. Mix gently, but thoroughly, to ensure that the solution is homogeneous.
3. Load the following volumes of the ladder on SDS-polyacrylamide gel:
   – 5 µL per well for mini gel,
   – 10 µL per well for large gel.
   Use the same volumes for Western blotting.
   The loading volumes listed above are recommended for gels with a thickness of 0.75-1.0 mm. The loading volume should be doubled for 1.5 mm thick gels.

Important Notes

- Prestained proteins can have different mobilities in various SDS-PAGE-buffer systems. However, they are suitable for approximate molecular weight determination when calibrated against unstained standards in the same system. See the table provided for migration patterns in different electrophoresis conditions.
- In low-percentage gels (< 10 %), the low-molecular weight proteins in the ladder may migrate with the dye front.
- PageRuler Plus Prestained Protein Ladder can be used in Western blotting with all common membranes: PVDF, nylon and nitrocellulose.
- Longer transfer times or higher transfer voltages may be required for Western blotting of large (>100 kDa) proteins.

PageRuler Plus Prestained Protein Ladder

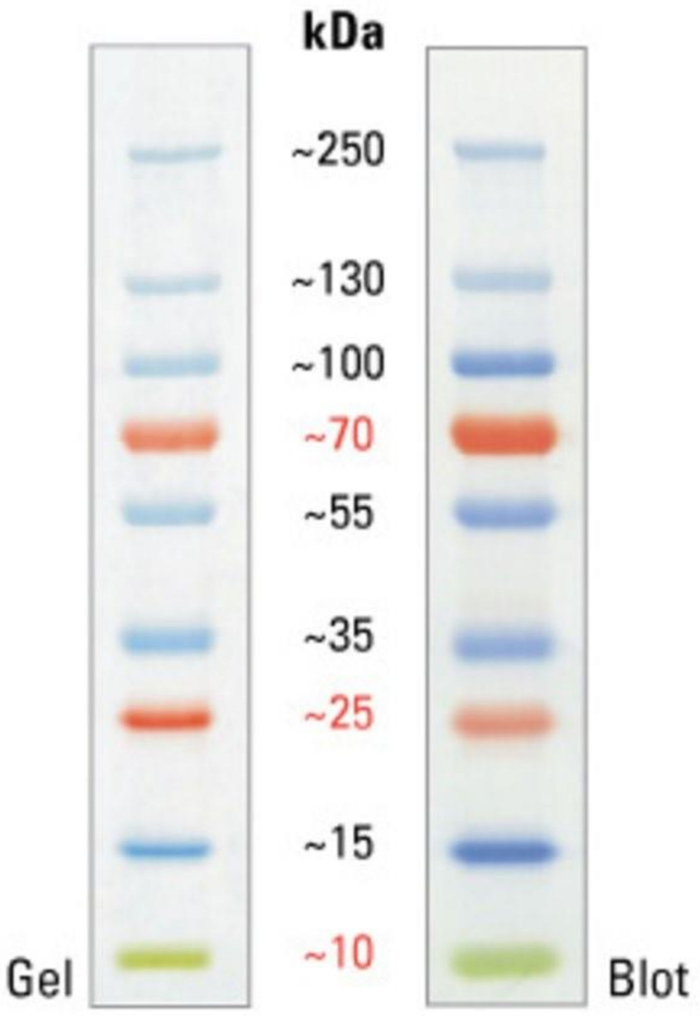

4-20% Tris-glycine SDS-PAGE

General References

Burnette, W.N. (1981). “Western blotting”: electrophoretic transfer of proteins from sodium dodecyl sulfate – polyacrylamide gels to unmodified nitrocellulose and radiographic detection with antibody and radioiodinated protein A. *Anal Biochem* 112(2):195-203.

Kurien, B.T. and Scofield, R.H. (2003). Protein blotting: a review. *J Imm Meth* 274:1-15.

Laemmli, U.K. (1970). Cleavage of structural proteins during the assembly of the head of bacteriophage T4. *Nature* 227:680-5.

Towbin, H., et al. (1979). Electrophoretic transfer of proteins from polyacrylamide gels to nitrocellulose sheets: procedure and some applications. *Proc Natl Acad Sci USA* 76:4350-4.

PRODUCT USE LIMITATION

This product is developed, designed and sold exclusively *for research purposes and in vitro use only.* The product was not tested for use in diagnostics or for drug development, nor is it suitable for administration to humans or animals.
Please refer to www.thermofisher.com for Material Safety Data Sheet of the product.

© 2019 Thermo Fisher Scientific Inc. All rights reserved. All trademarks are the property of Thermo Fisher Scientific Inc. and its subsidiaries.
