# Supplementary figures and images for: Streptococcus pyogenes EVs induce the alternative inflammasome via caspase-4/-5 in human monocytes
Source: EMBO Rep. 2025 Sep 8;26(19):4847–85. doi: 10.1038/s44319-025-00558-7 (PMC12508482; doi:10.1038/s44319-025-00558-7)

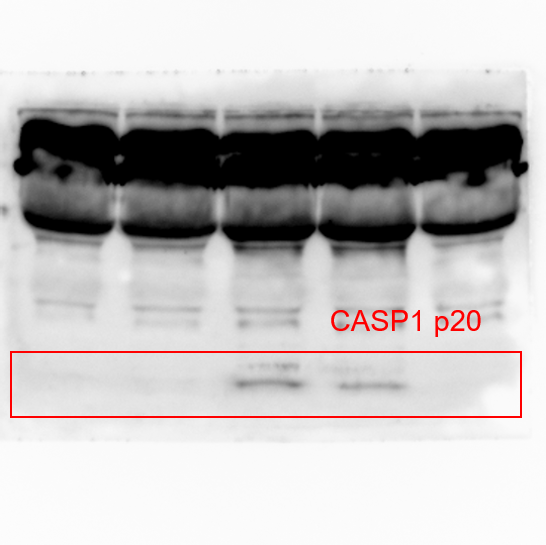

Supplement: Supplementary file 5 — Source data Fig. 2 [file 44319_2025_558_MOESM5_ESM.zip › Figure_2/Figure_2E/Macrophages_CASP1.tif]

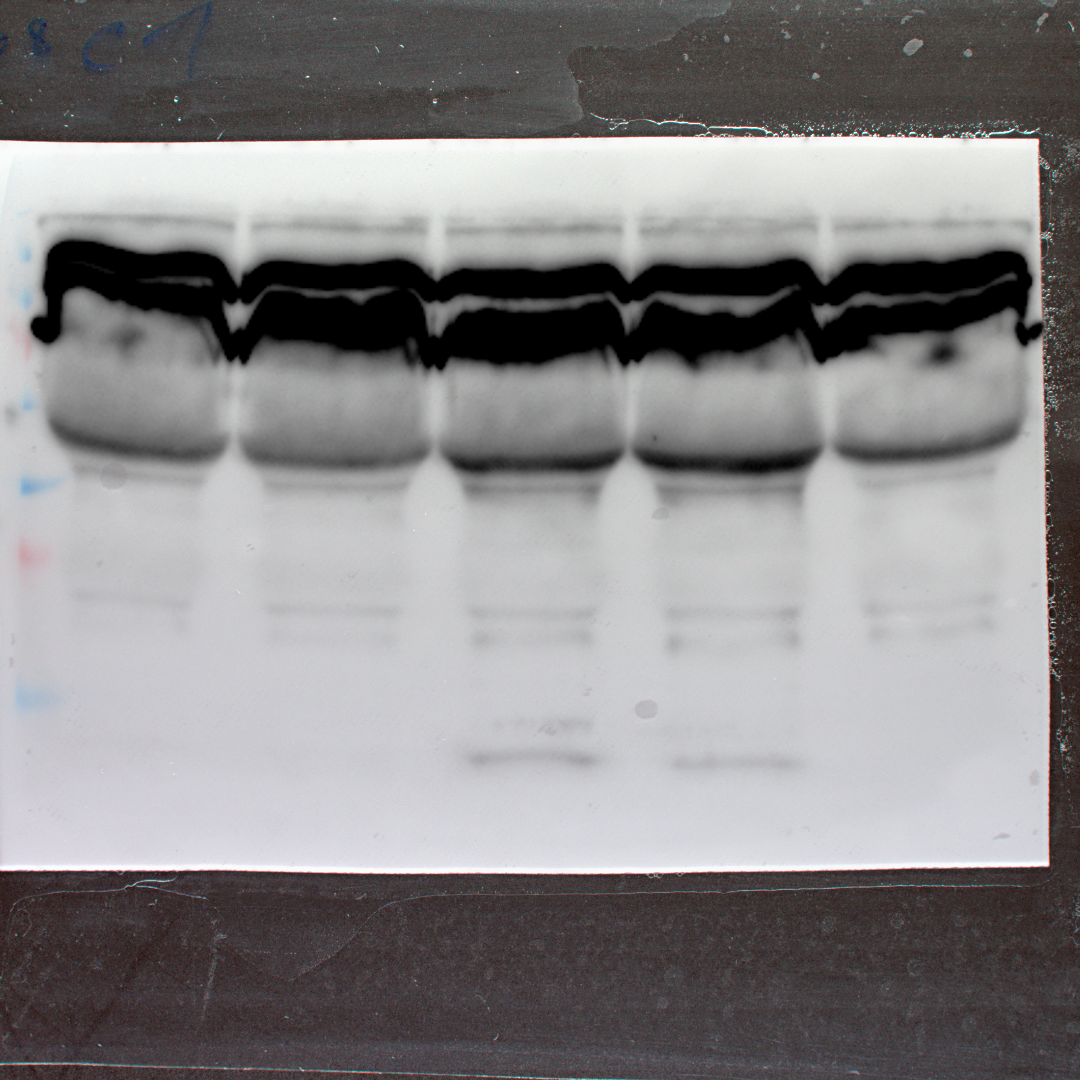

Supplement: Supplementary file 5 — Source data Fig. 2 [file 44319_2025_558_MOESM5_ESM.zip › Figure_2/Figure_2E/Macrophages_CASP1_ladder.Tif]

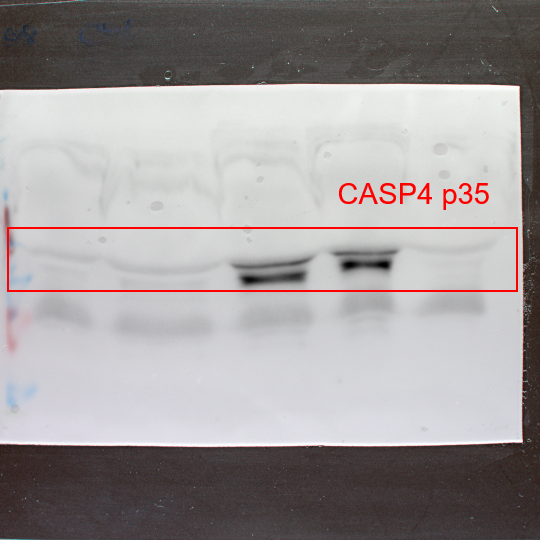

Supplement: Supplementary file 5 — Source data Fig. 2 [file 44319_2025_558_MOESM5_ESM.zip › Figure_2/Figure_2E/Macrophages_CASP4_ladder.tif]

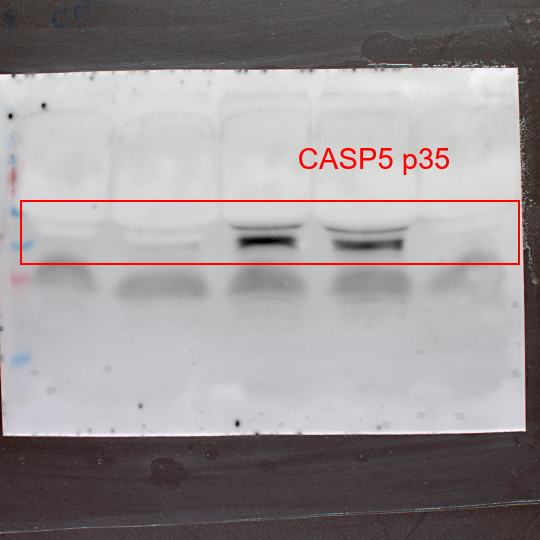

Supplement: Supplementary file 5 — Source data Fig. 2 [file 44319_2025_558_MOESM5_ESM.zip › Figure_2/Figure_2E/Macrophages_CASP5_ladder.tif]

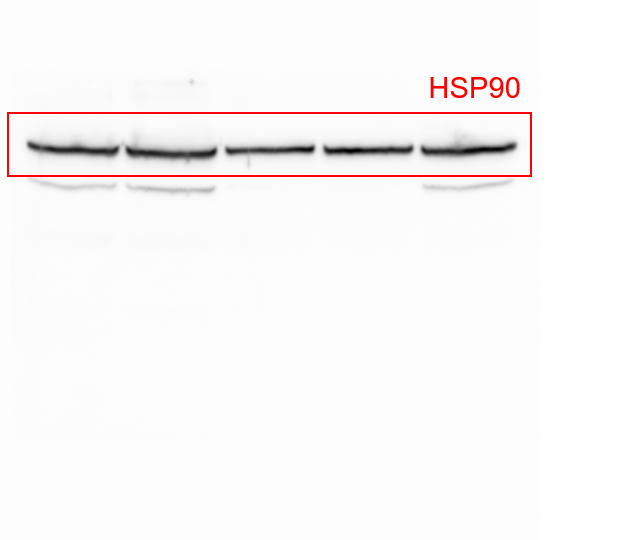

Supplement: Supplementary file 5 — Source data Fig. 2 [file 44319_2025_558_MOESM5_ESM.zip › Figure_2/Figure_2E/Macrophages_HSP90.tif]

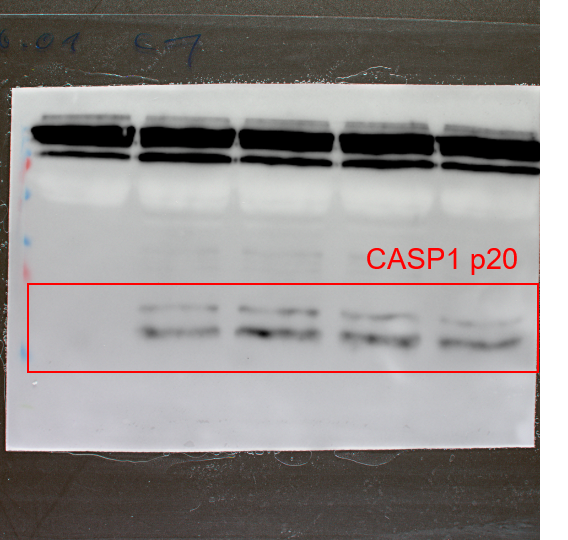

Supplement: Supplementary file 5 — Source data Fig. 2 [file 44319_2025_558_MOESM5_ESM.zip › Figure_2/Figure_2E/Monocytes_CASP1_ladder.tif]

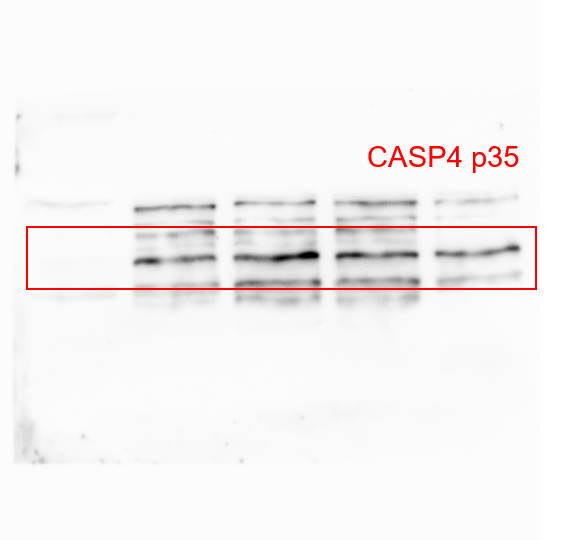

Supplement: Supplementary file 5 — Source data Fig. 2 [file 44319_2025_558_MOESM5_ESM.zip › Figure_2/Figure_2E/Monocytes_CASP4.tif]

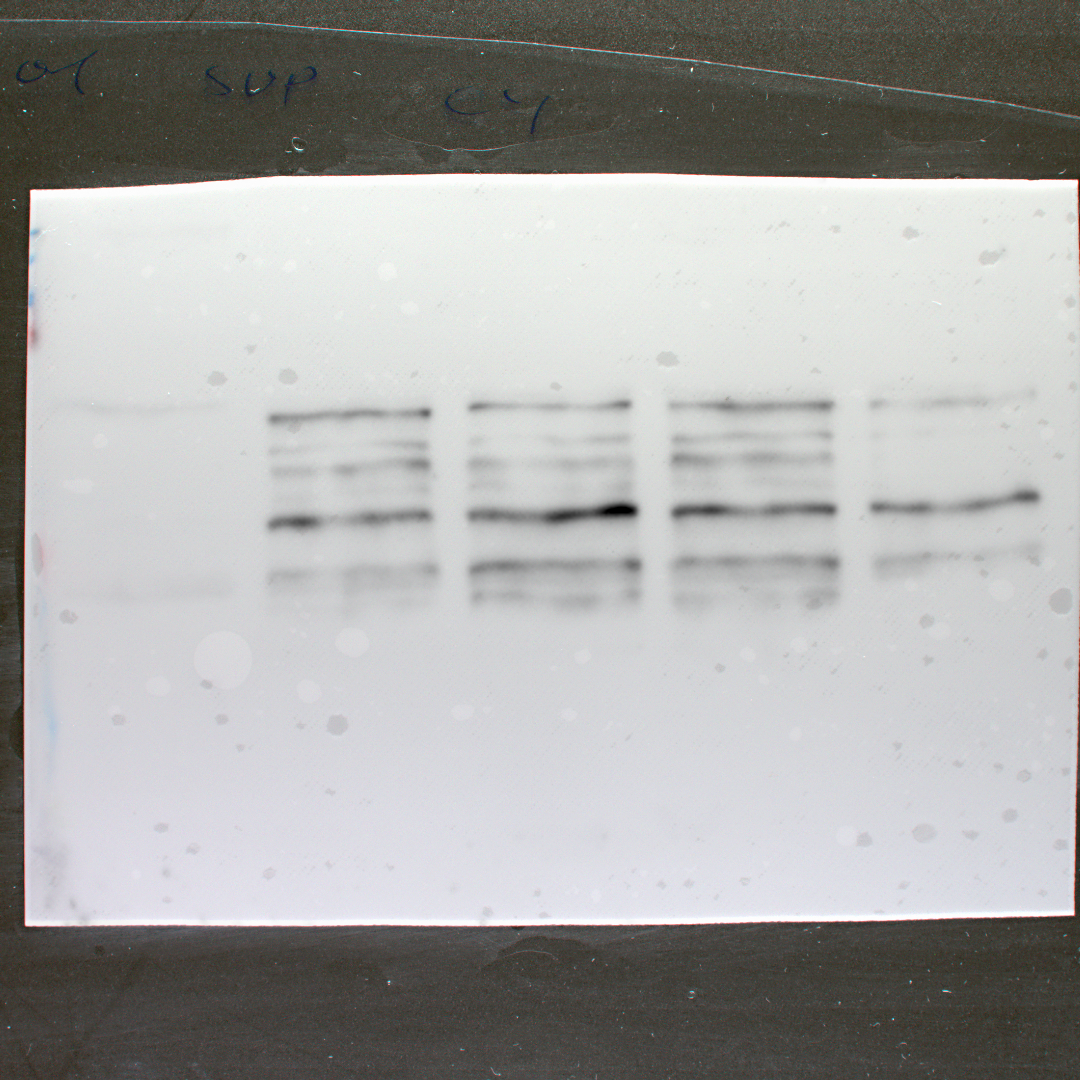

Supplement: Supplementary file 5 — Source data Fig. 2 [file 44319_2025_558_MOESM5_ESM.zip › Figure_2/Figure_2E/Monocytes_CASP4_ladder.Tif]

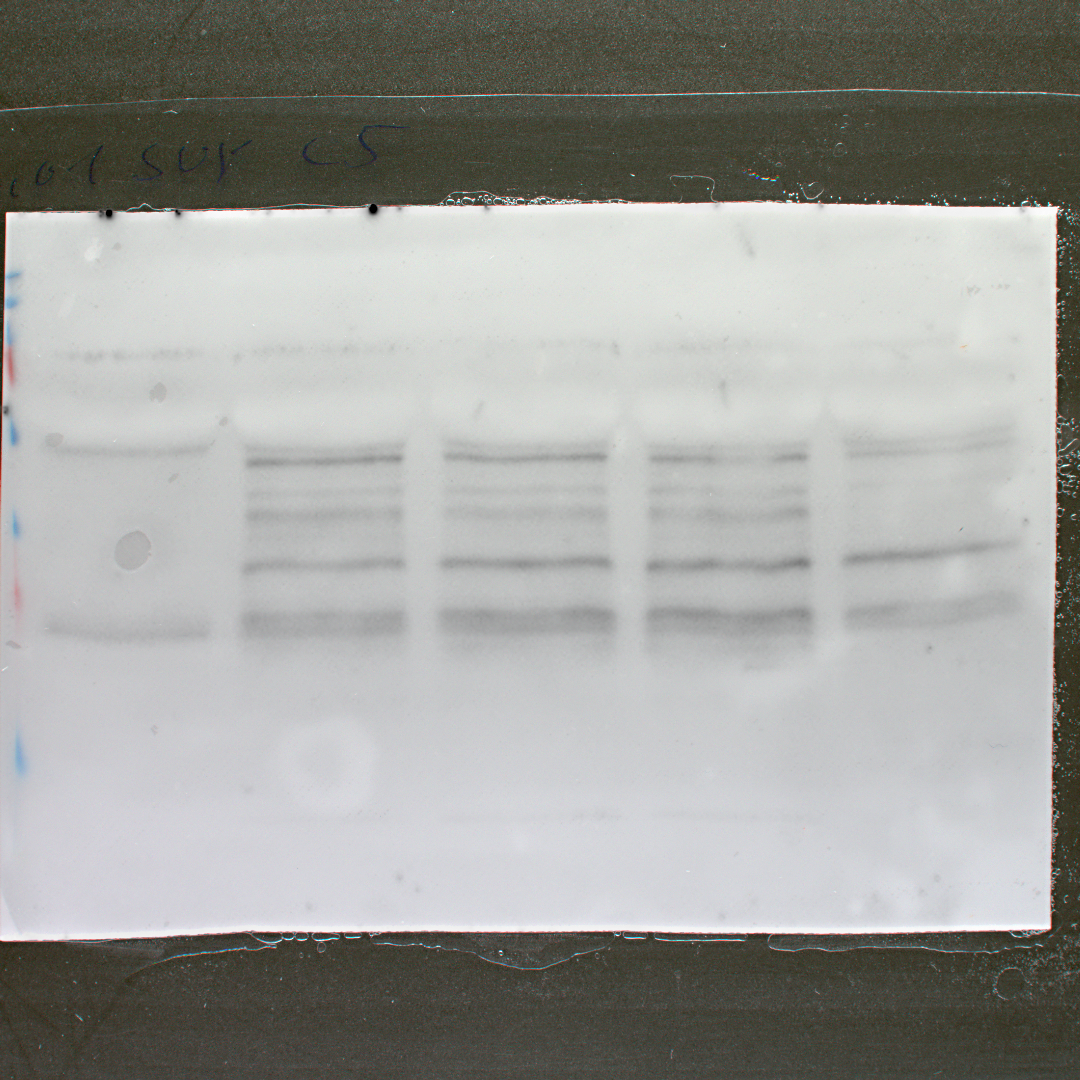

Supplement: Supplementary file 5 — Source data Fig. 2 [file 44319_2025_558_MOESM5_ESM.zip › Figure_2/Figure_2E/Monocytes_CASP5_ladder.Tif]

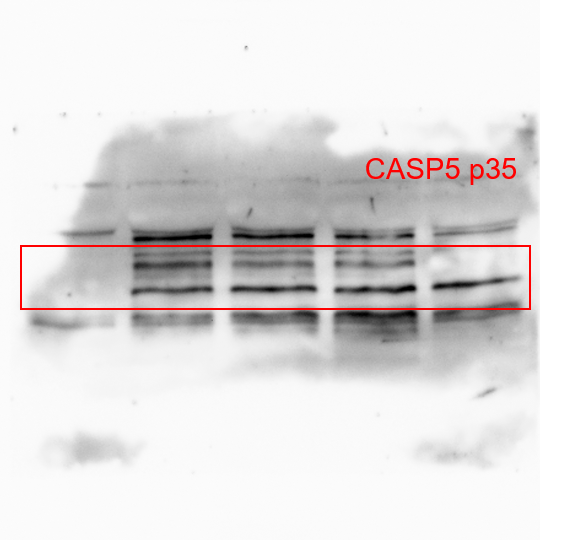

Supplement: Supplementary file 5 — Source data Fig. 2 [file 44319_2025_558_MOESM5_ESM.zip › Figure_2/Figure_2E/Monocytes_CASP5_reprobe.tif]

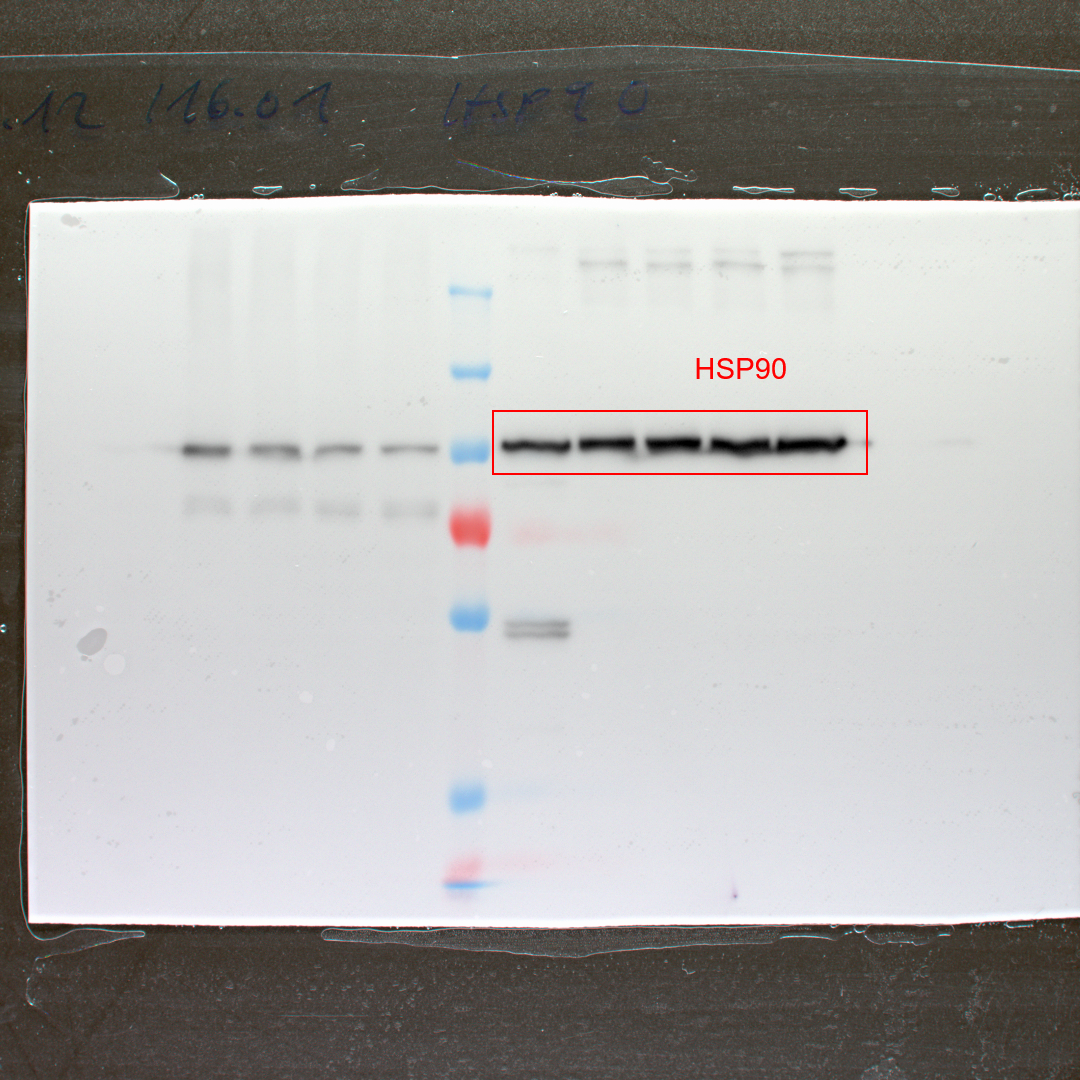

Supplement: Supplementary file 5 — Source data Fig. 2 [file 44319_2025_558_MOESM5_ESM.zip › Figure_2/Figure_2E/Monocytes_HSP90.tif]

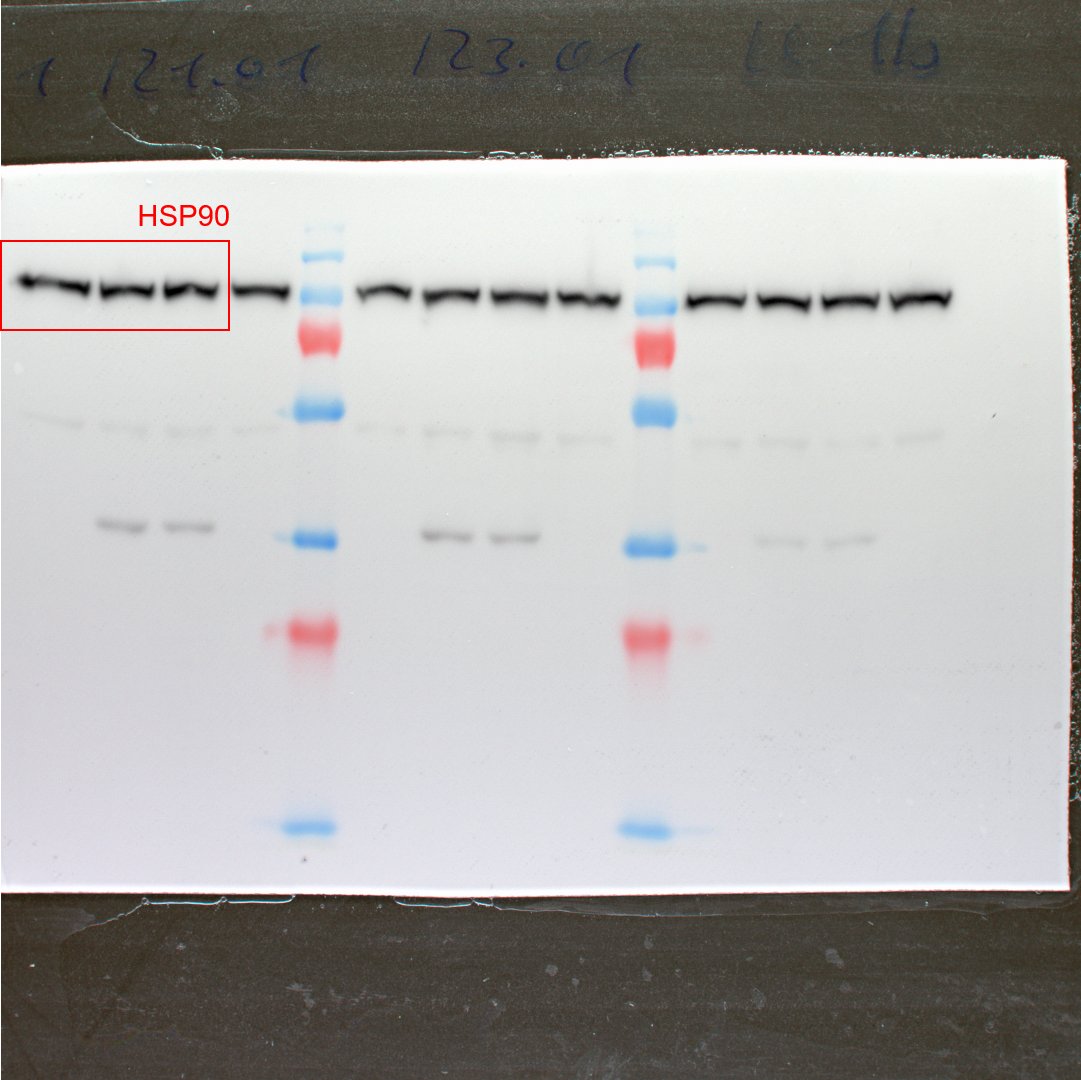

Supplement: Supplementary file 5 — Source data Fig. 2 [file 44319_2025_558_MOESM5_ESM.zip › Figure_2/Figure_2F/1_HSP90_2h_ladder.tif]

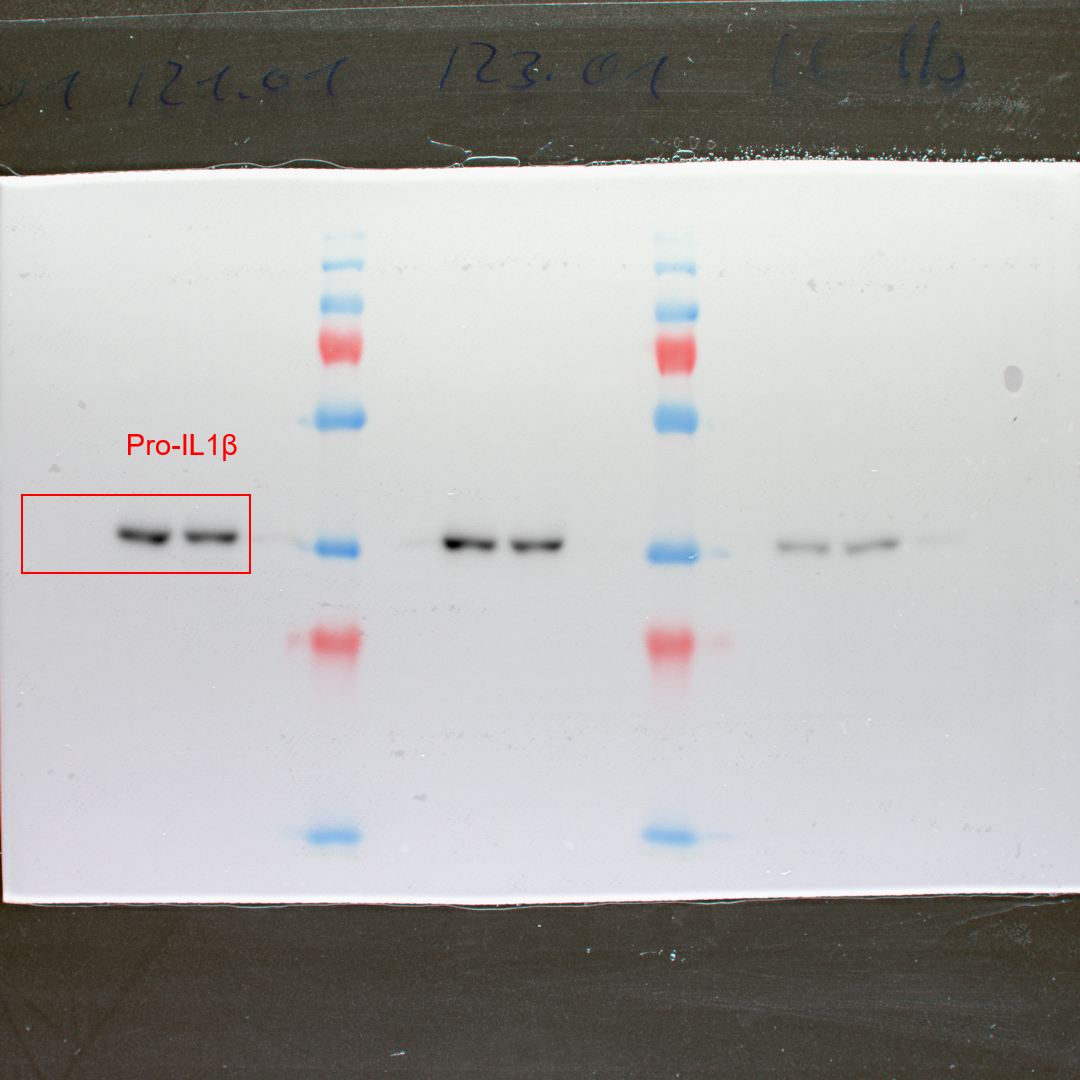

Supplement: Supplementary file 5 — Source data Fig. 2 [file 44319_2025_558_MOESM5_ESM.zip › Figure_2/Figure_2F/1_Pro_IL1B_2h_ladder.tif]

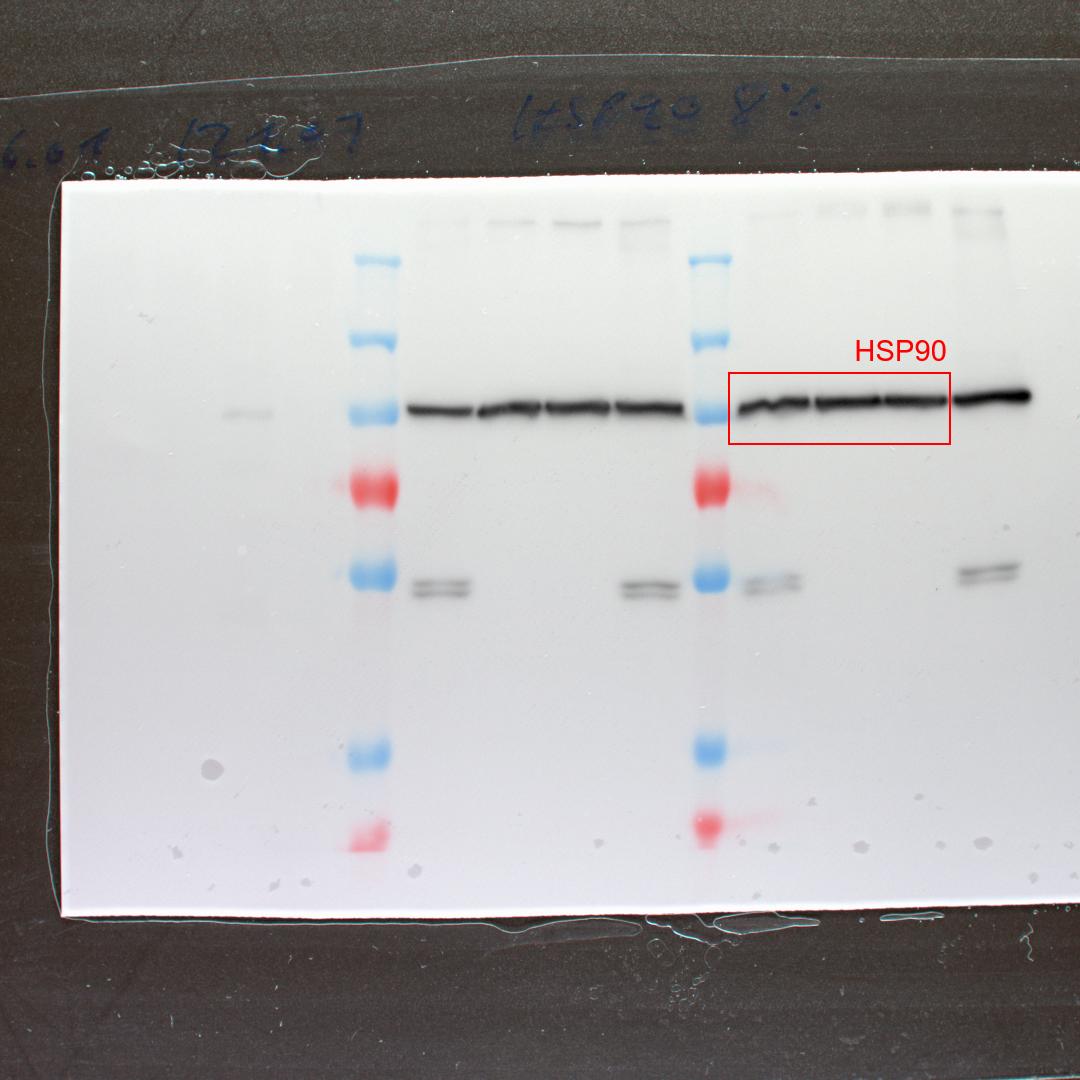

Supplement: Supplementary file 5 — Source data Fig. 2 [file 44319_2025_558_MOESM5_ESM.zip › Figure_2/Figure_2F/2_HSP90_18h_ladder.tif]

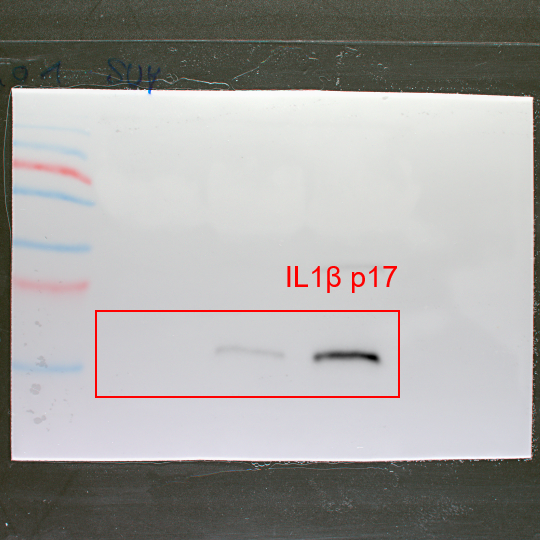

Supplement: Supplementary file 5 — Source data Fig. 2 [file 44319_2025_558_MOESM5_ESM.zip › Figure_2/Figure_2F/2_IL1B_18h_ladder.tif]

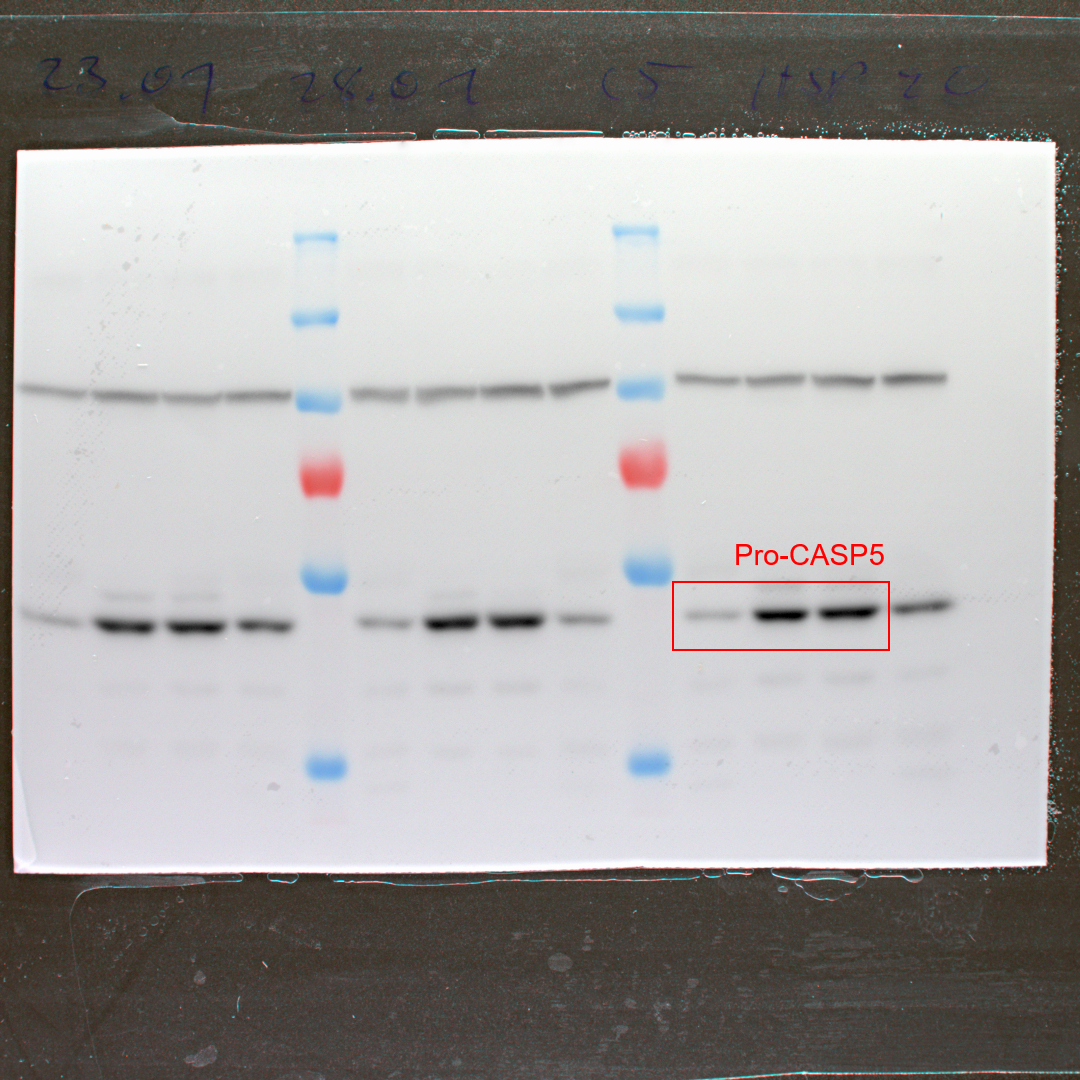

Supplement: Supplementary file 5 — Source data Fig. 2 [file 44319_2025_558_MOESM5_ESM.zip › Figure_2/Figure_2F/3_CASP5_18h_ladder.tif]

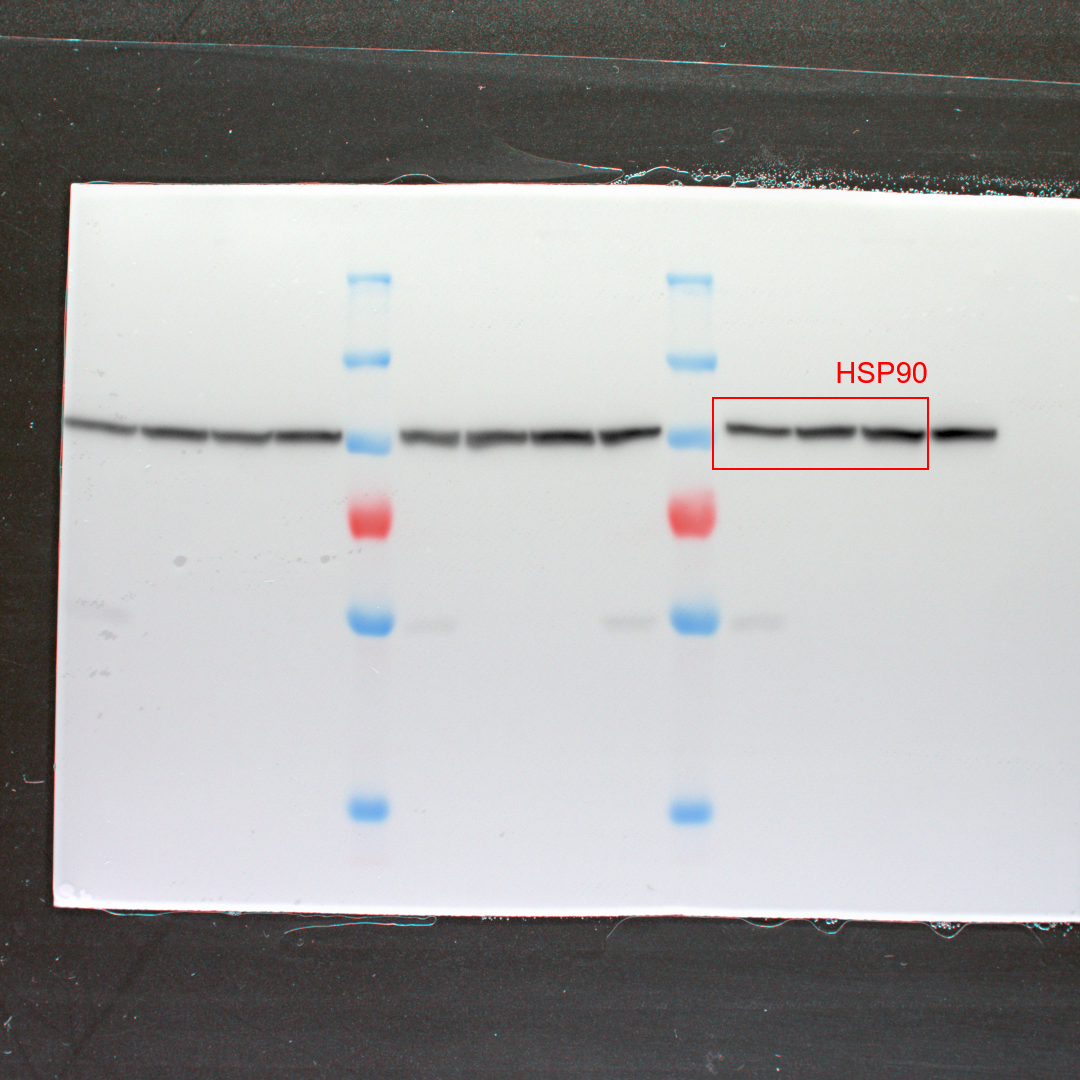

Supplement: Supplementary file 5 — Source data Fig. 2 [file 44319_2025_558_MOESM5_ESM.zip › Figure_2/Figure_2F/3_HSP90_18h_ladder.tif]

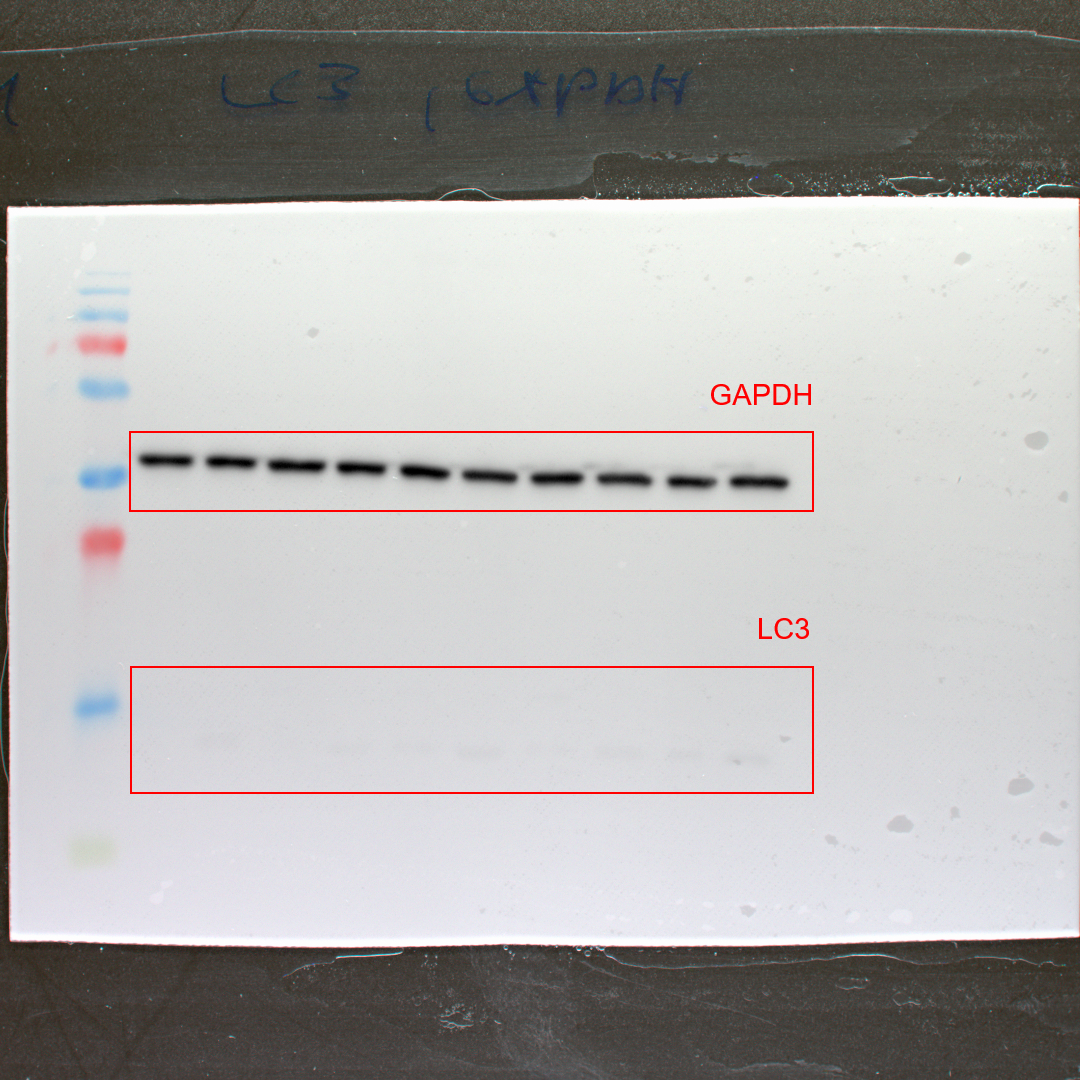

Supplement: Supplementary file 5 — Source data Fig. 2 [file 44319_2025_558_MOESM5_ESM.zip › Figure_2/Figure_2G/Monocytes_GAPDH_LC3_ladder.tif]

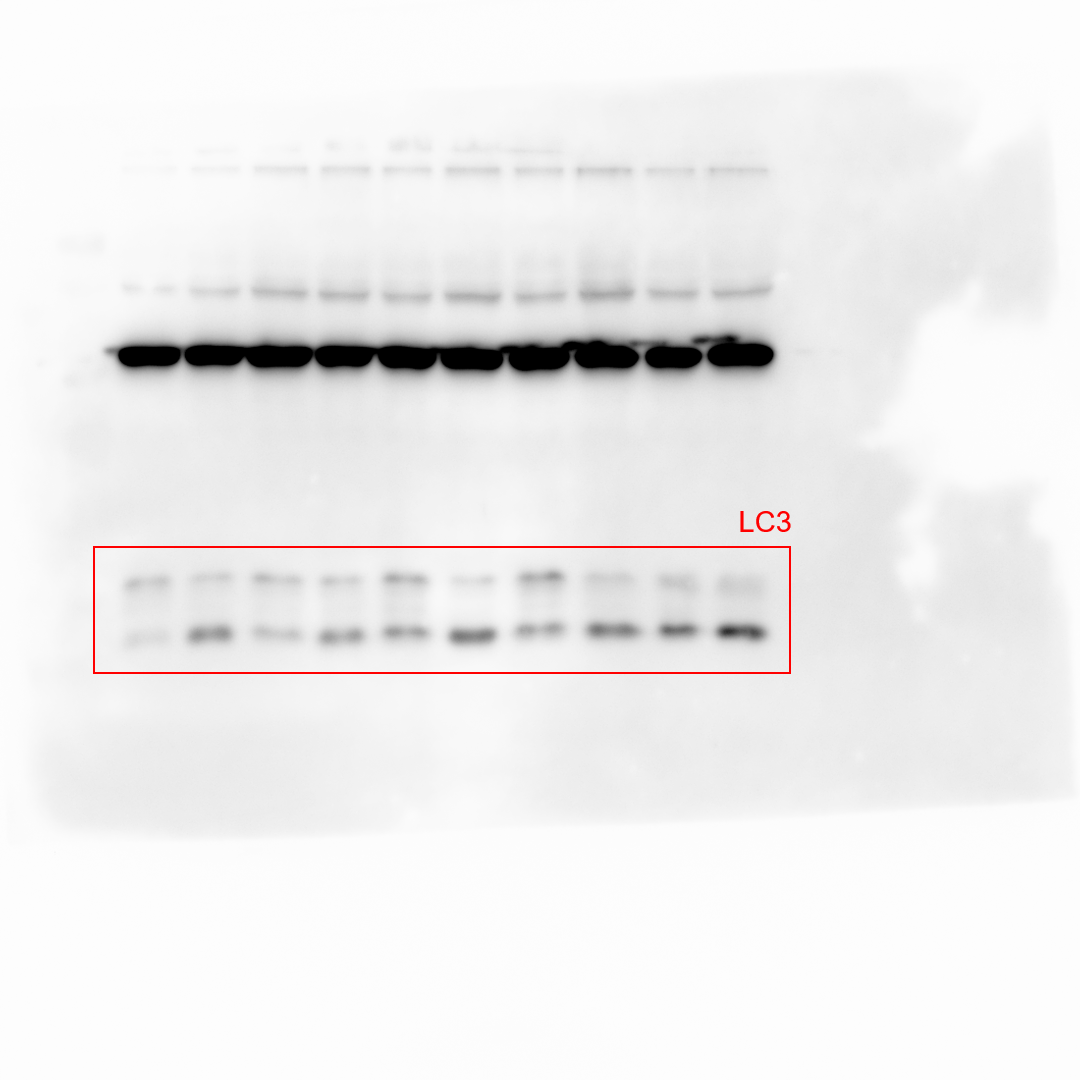

Supplement: Supplementary file 5 — Source data Fig. 2 [file 44319_2025_558_MOESM5_ESM.zip › Figure_2/Figure_2G/Monocytes_GAPDH_LC3_longer_exposure.tif]

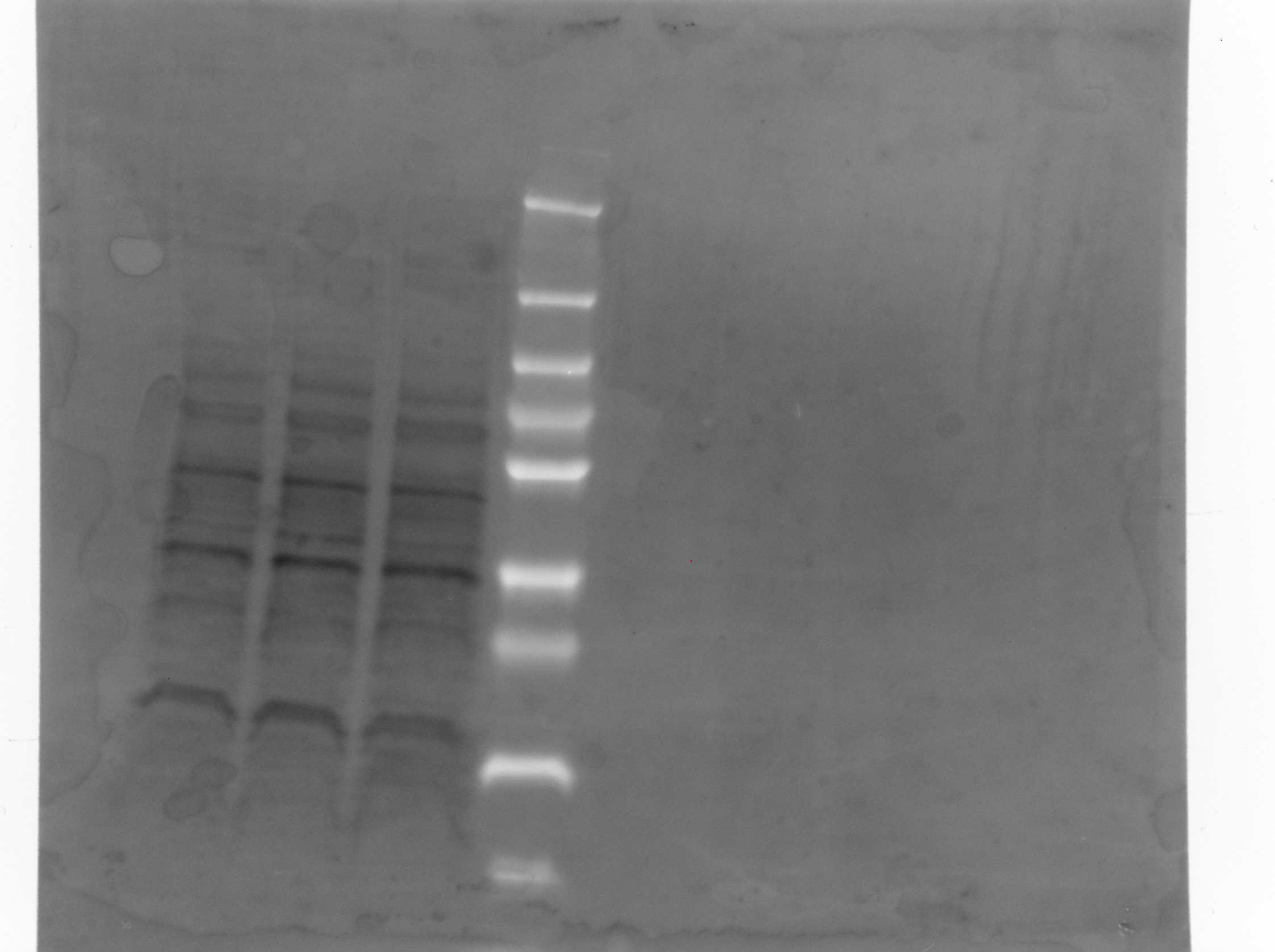

Supplement: Supplementary file 8 — Source data Fig. 5 [file 44319_2025_558_MOESM8_ESM.zip › Figure_5/Figure_5C/EV_batches_no_stain.tif]

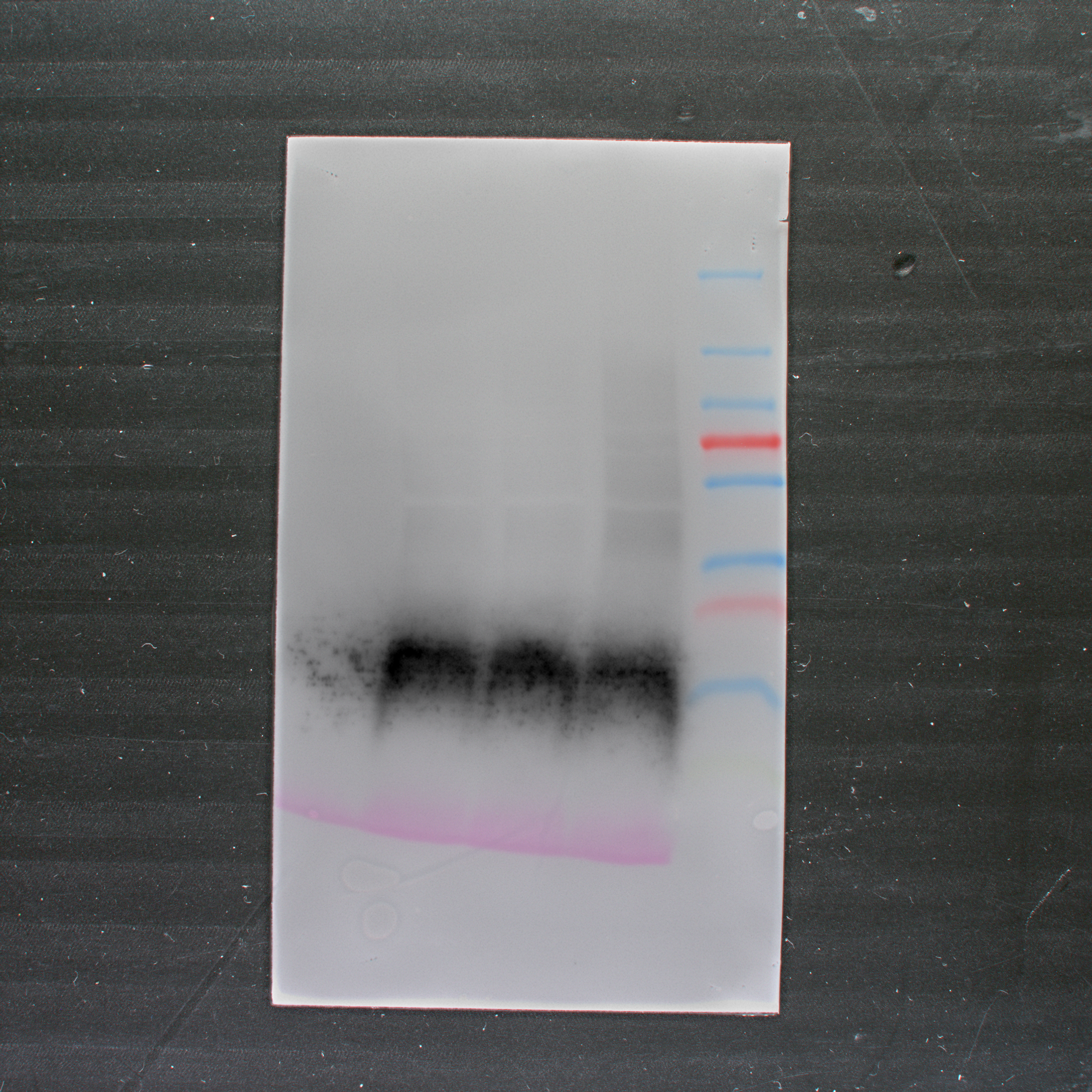

Supplement: Supplementary file 8 — Source data Fig. 5 [file 44319_2025_558_MOESM8_ESM.zip › Figure_5/Figure_5C/LTA_EV_batches_ladder.Tif]

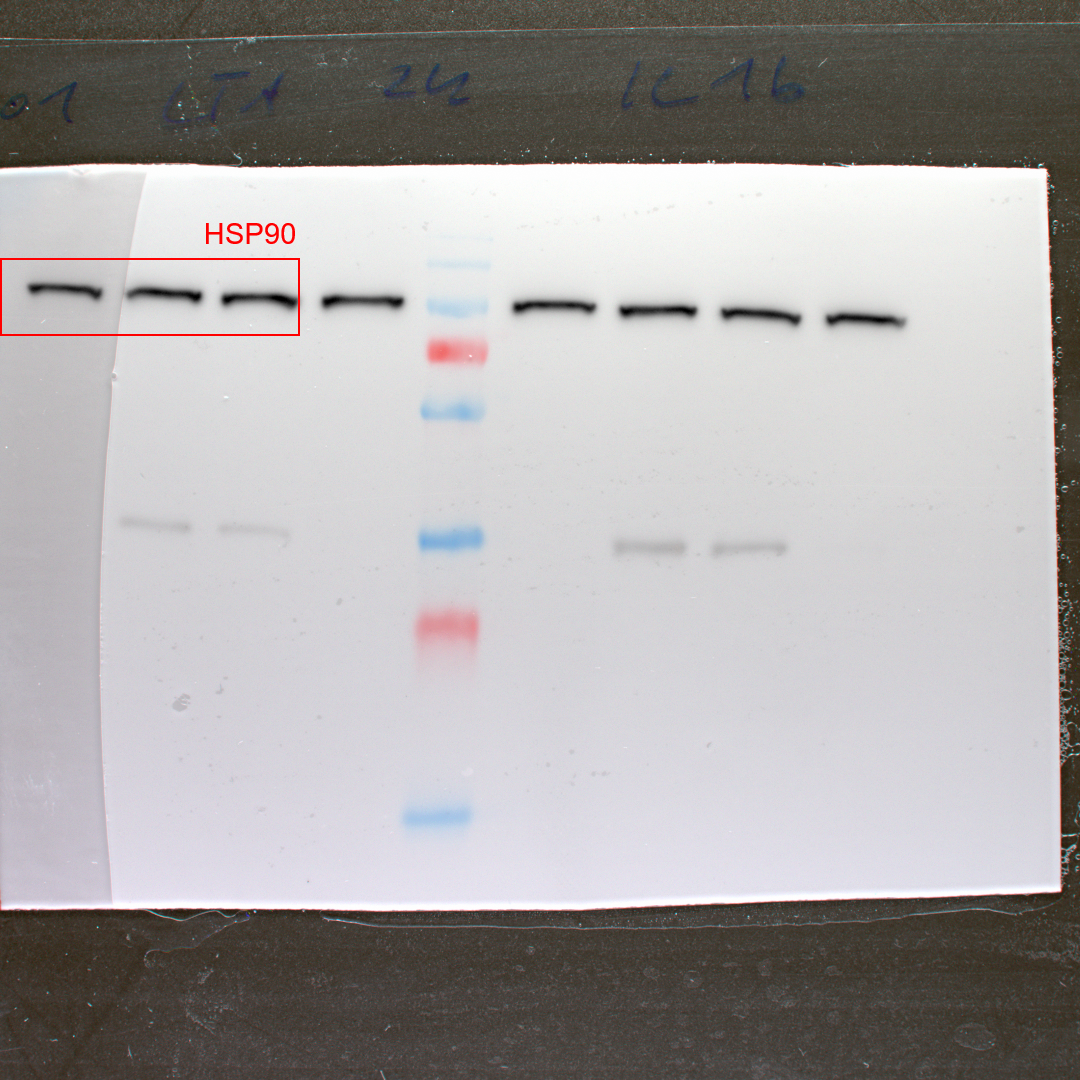

Supplement: Supplementary file 8 — Source data Fig. 5 [file 44319_2025_558_MOESM8_ESM.zip › Figure_5/Figure_5H/1_HSP90_2h_ladder.tif]

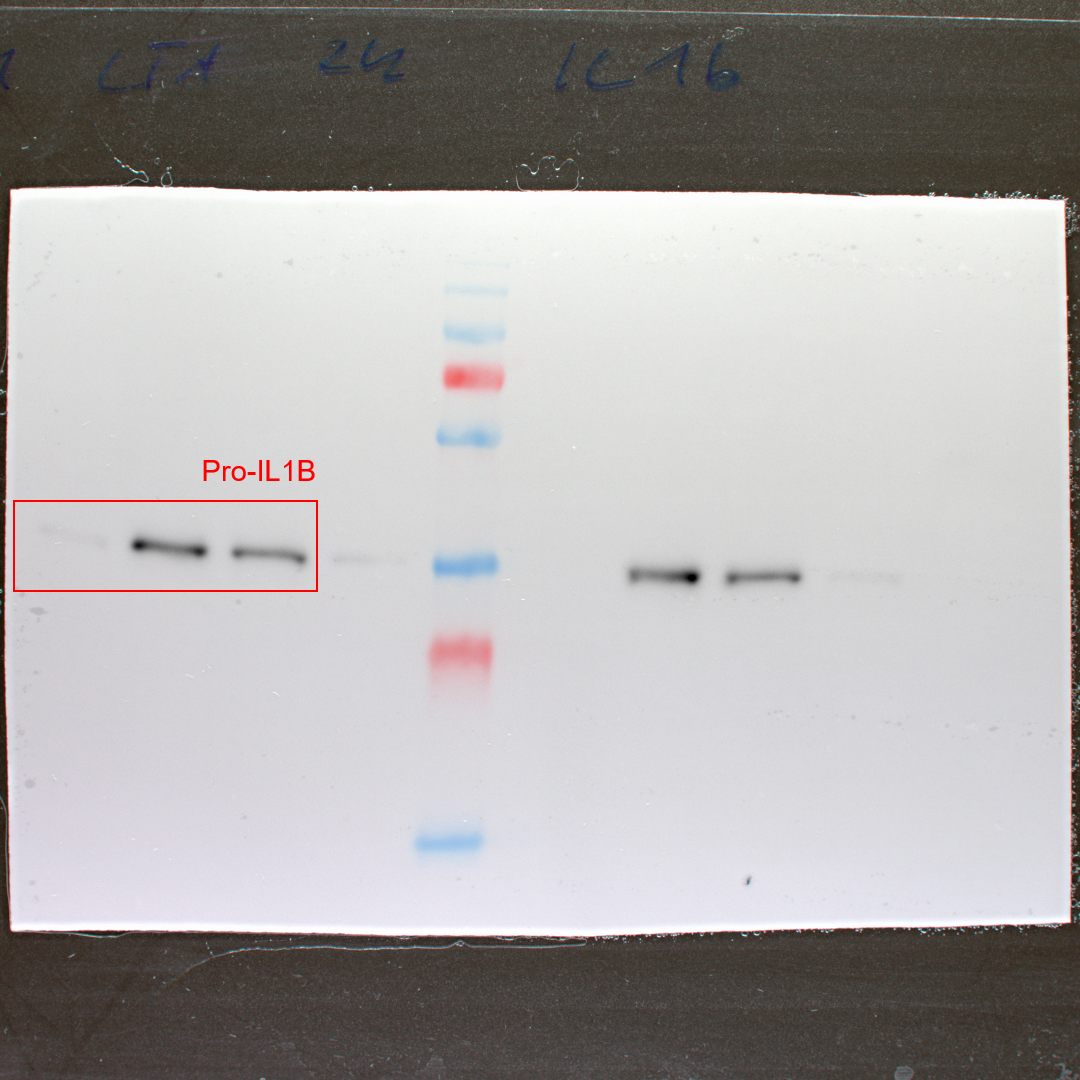

Supplement: Supplementary file 8 — Source data Fig. 5 [file 44319_2025_558_MOESM8_ESM.zip › Figure_5/Figure_5H/1_IL1B_2h_ladder.tif]

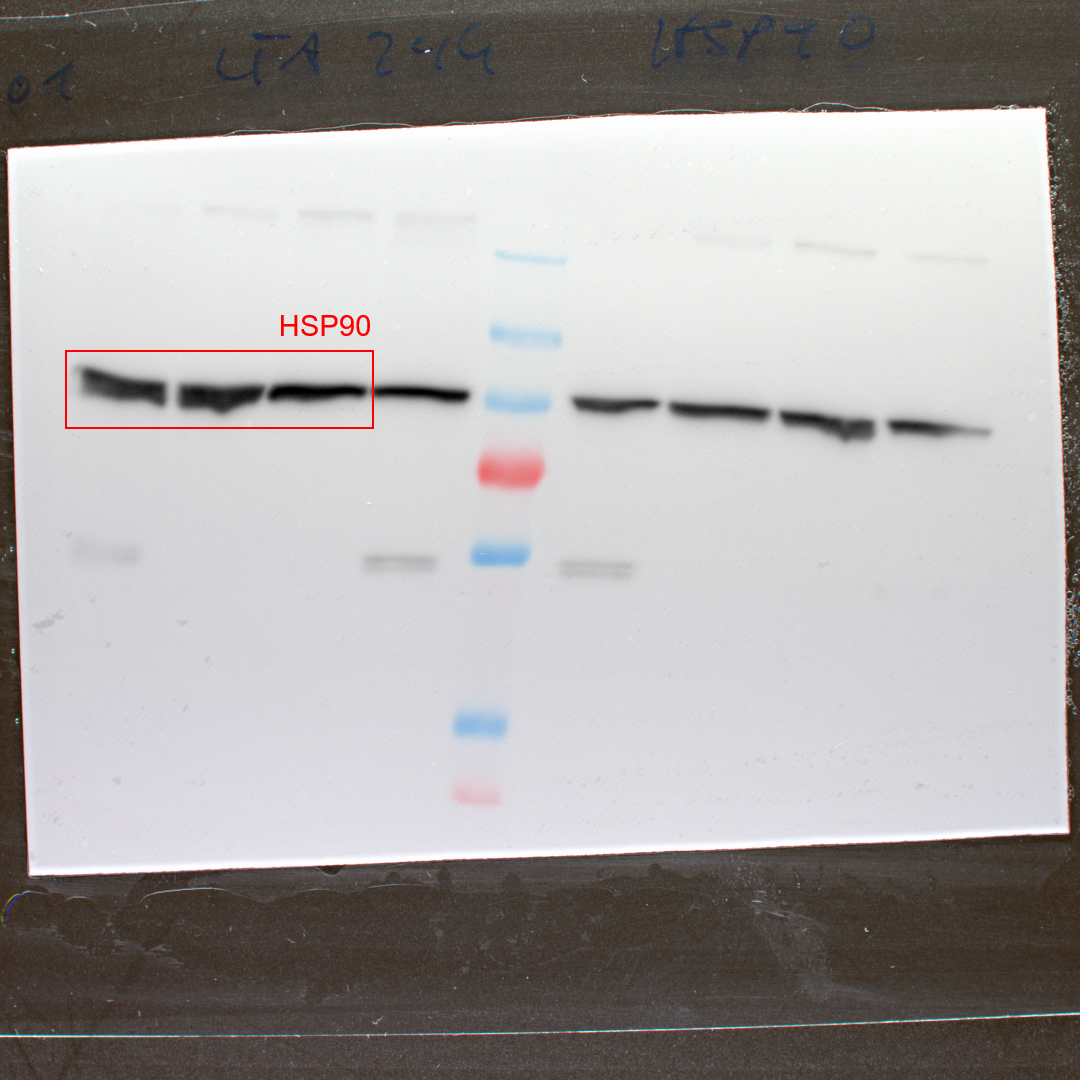

Supplement: Supplementary file 8 — Source data Fig. 5 [file 44319_2025_558_MOESM8_ESM.zip › Figure_5/Figure_5H/2_HSP90_18h_ladder.tif]

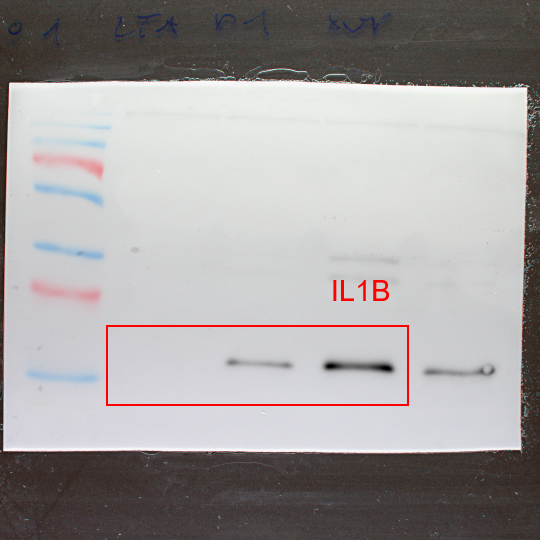

Supplement: Supplementary file 8 — Source data Fig. 5 [file 44319_2025_558_MOESM8_ESM.zip › Figure_5/Figure_5H/2_IL1B_18h_ladder.tif]

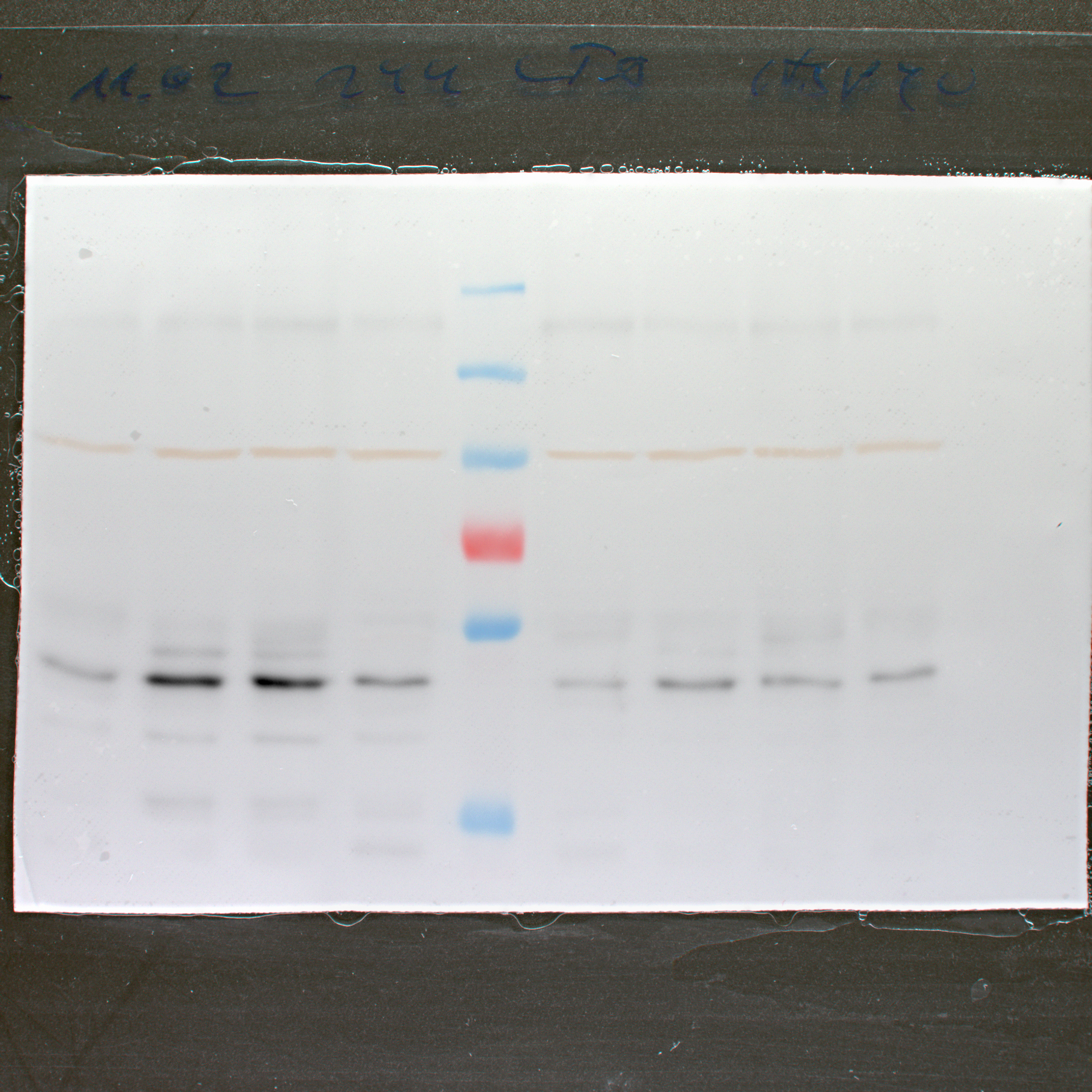

Supplement: Supplementary file 8 — Source data Fig. 5 [file 44319_2025_558_MOESM8_ESM.zip › Figure_5/Figure_5H/3_CASP5_18h_ladder.Tif]

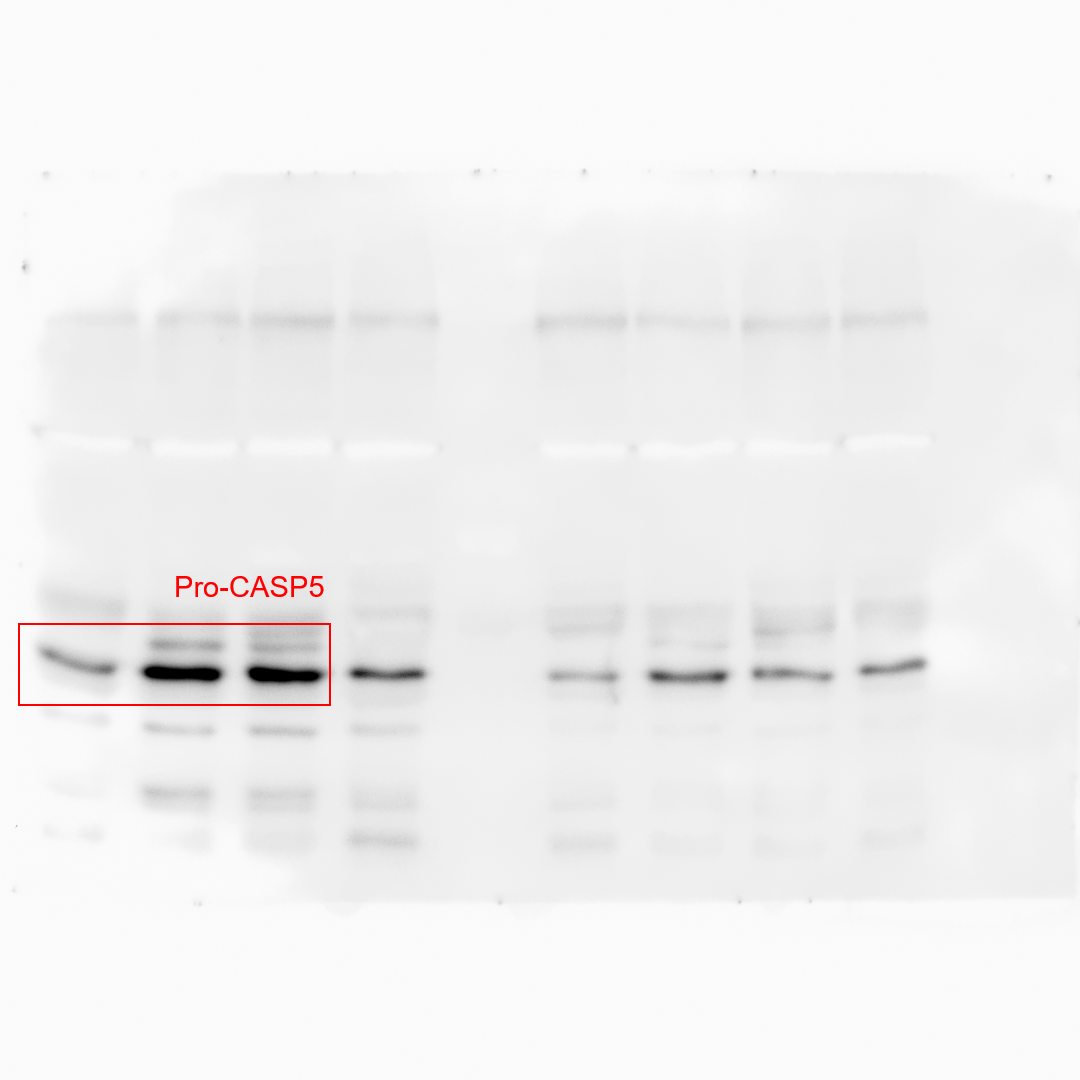

Supplement: Supplementary file 8 — Source data Fig. 5 [file 44319_2025_558_MOESM8_ESM.zip › Figure_5/Figure_5H/3_CASP5_18h_longer_exposure.tif]

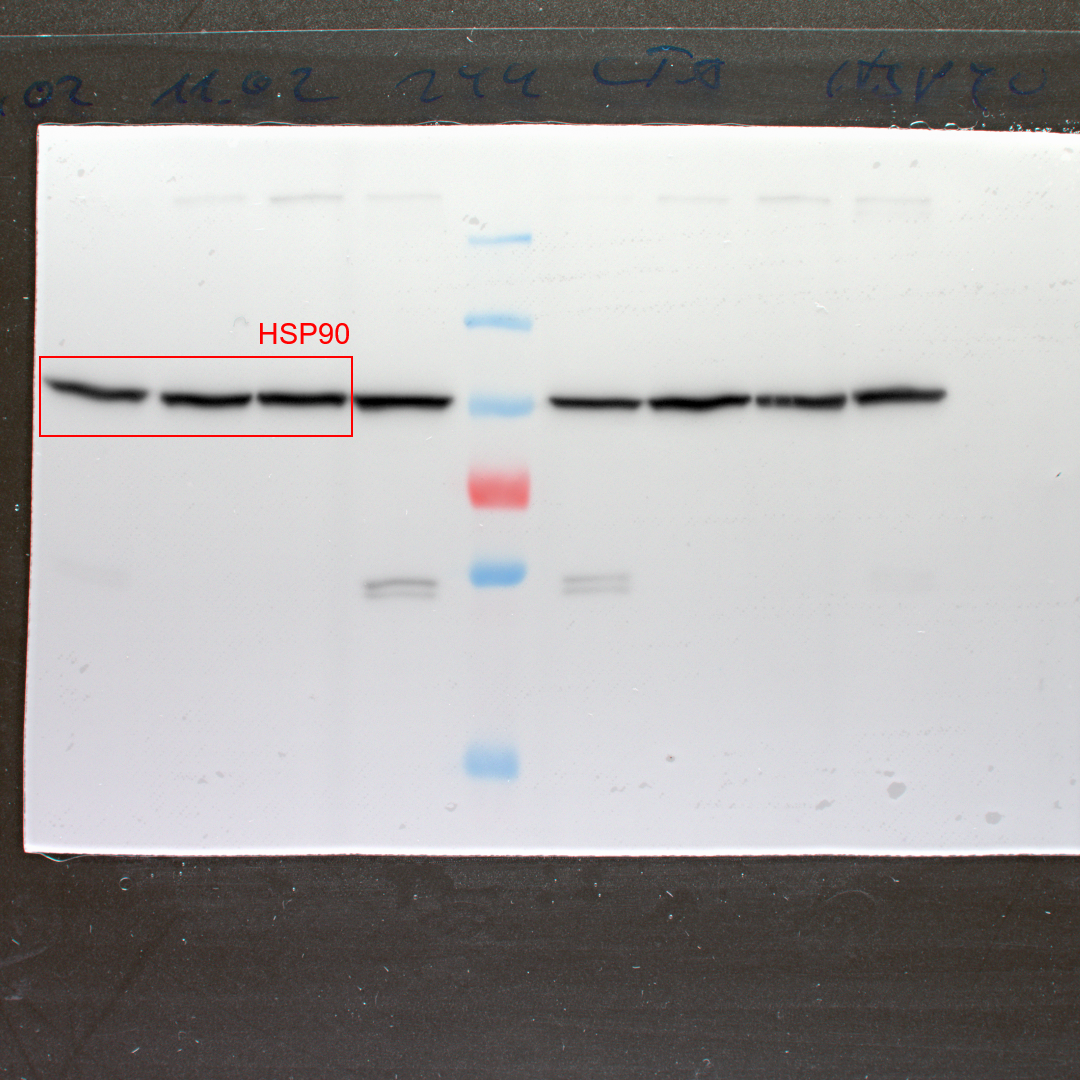

Supplement: Supplementary file 8 — Source data Fig. 5 [file 44319_2025_558_MOESM8_ESM.zip › Figure_5/Figure_5H/3_HSP90_18h_ladder.tif]

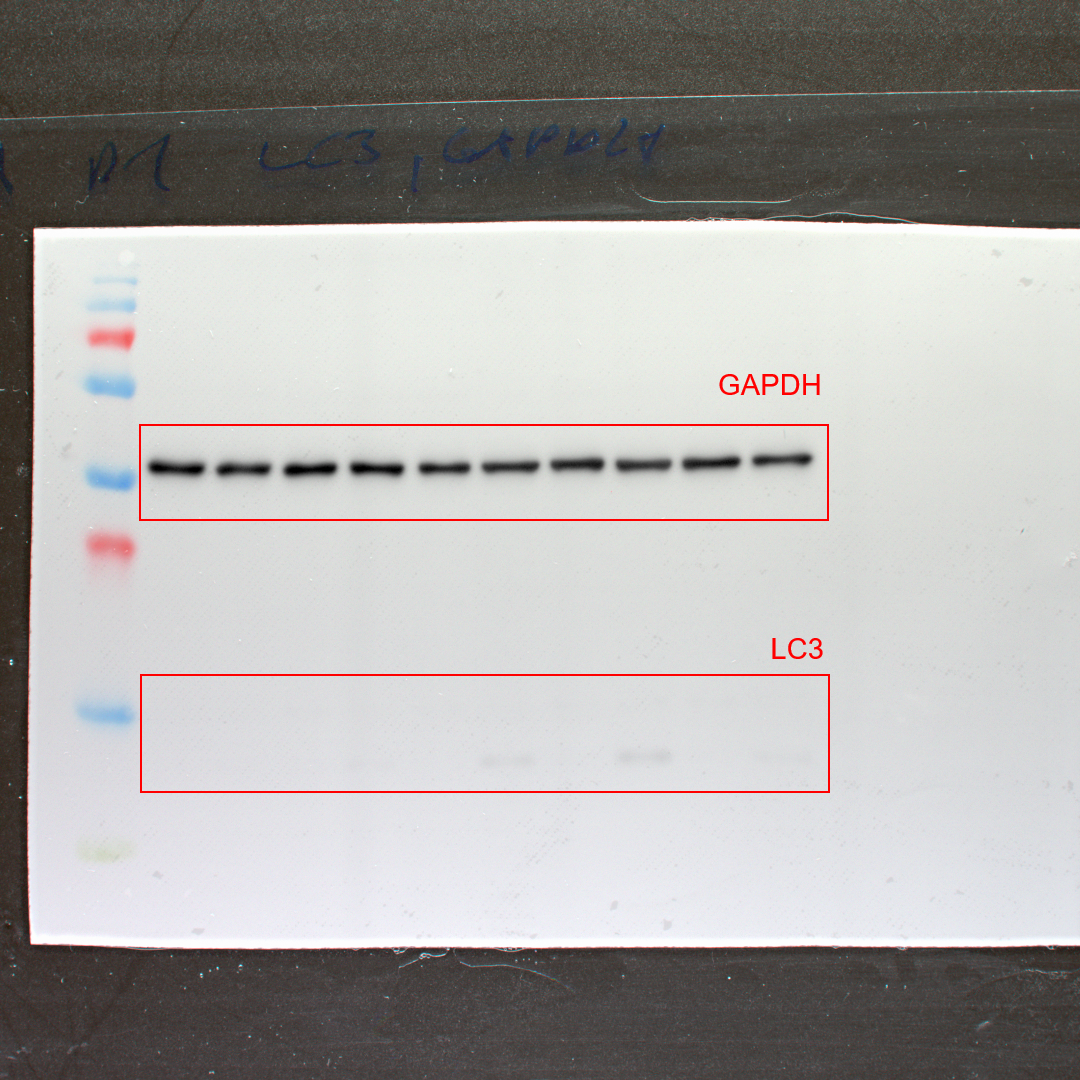

Supplement: Supplementary file 8 — Source data Fig. 5 [file 44319_2025_558_MOESM8_ESM.zip › Figure_5/Figure_5I/GAPDH_LC3_ladder.tif]

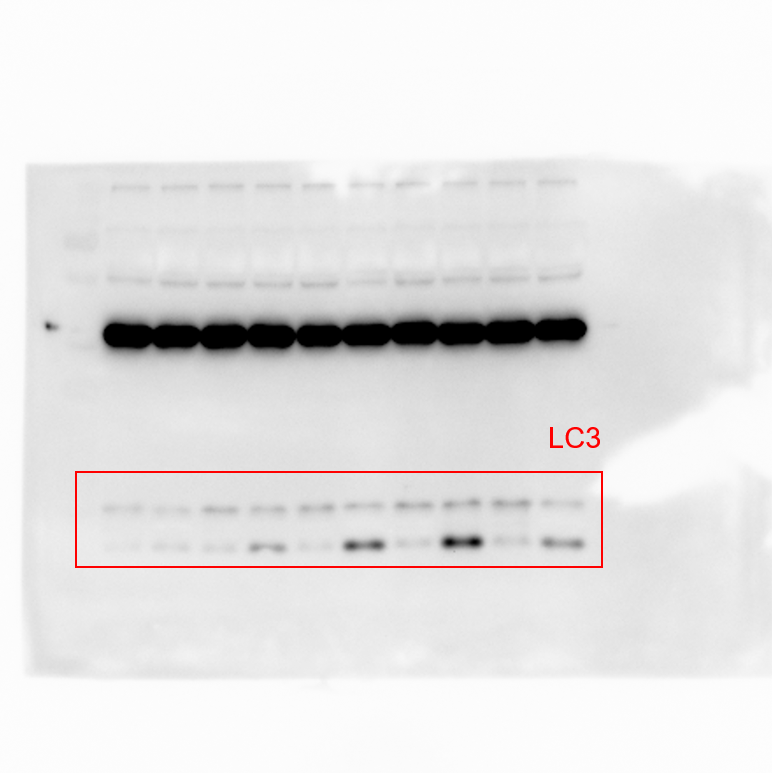

Supplement: Supplementary file 8 — Source data Fig. 5 [file 44319_2025_558_MOESM8_ESM.zip › Figure_5/Figure_5I/GAPDH_LC3_longer_exposure.tif]

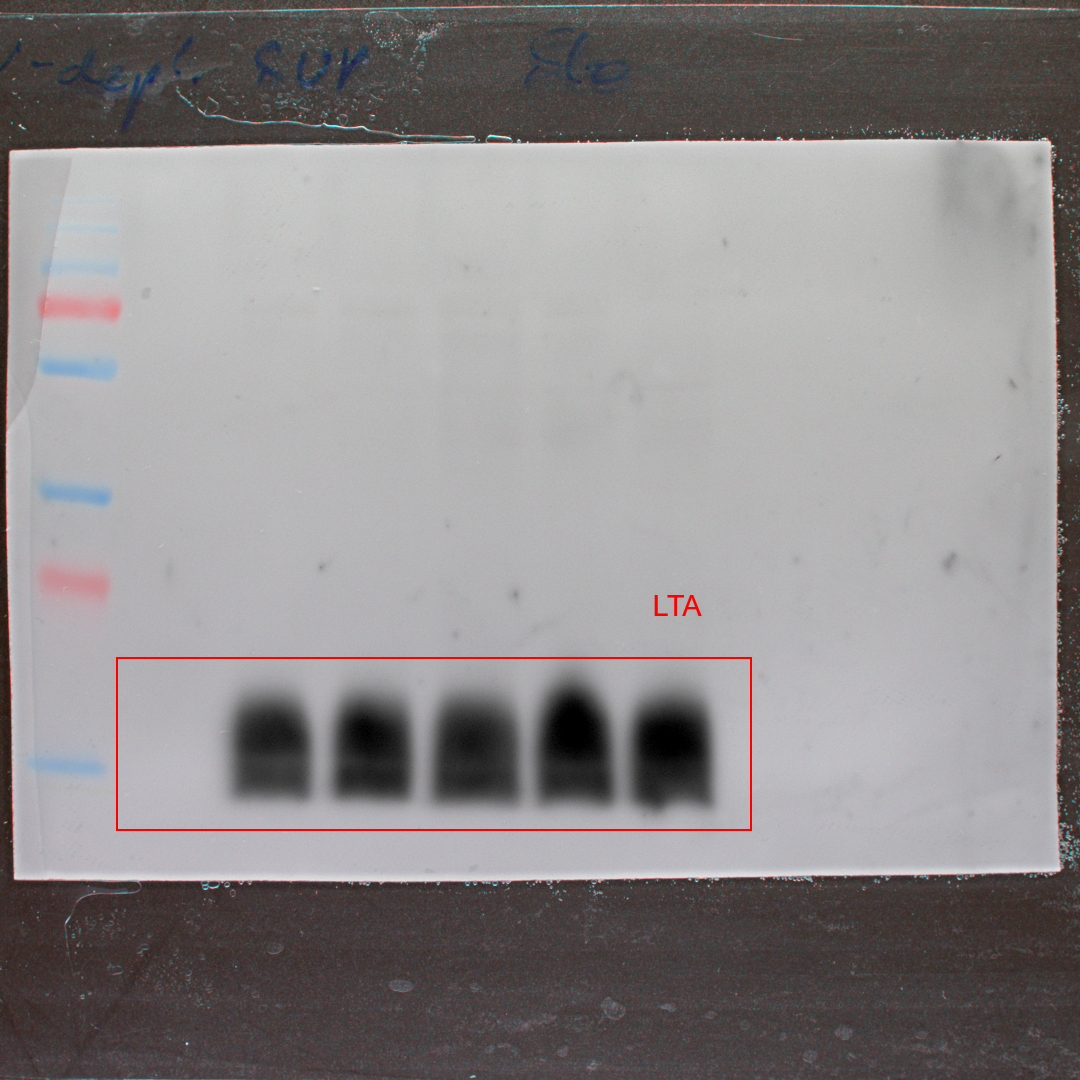

Supplement: Supplementary file 9 — Source data Fig. 6 [file 44319_2025_558_MOESM9_ESM.zip › Figure_6/Figure_6C/LTA_ladder.tif]

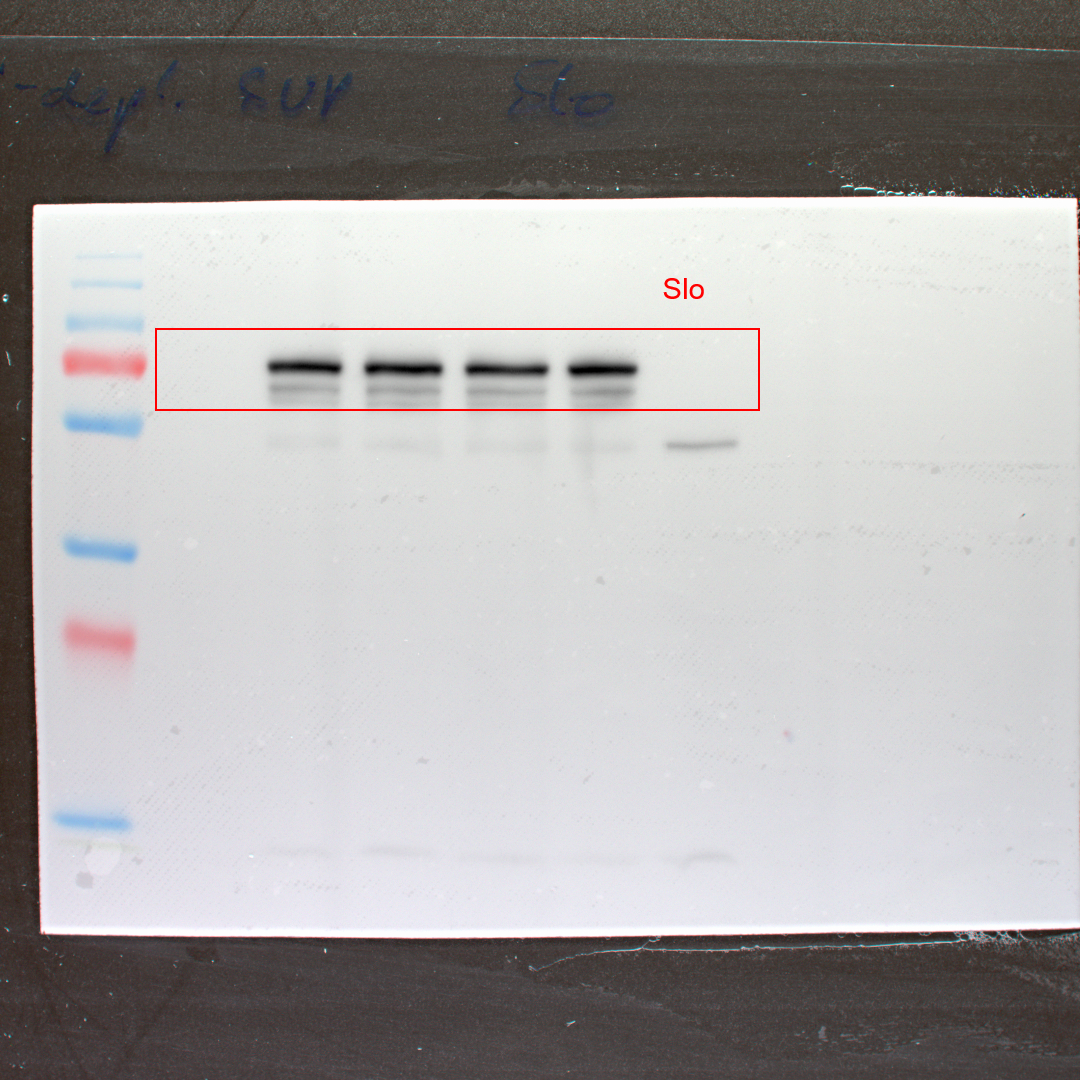

Supplement: Supplementary file 9 — Source data Fig. 6 [file 44319_2025_558_MOESM9_ESM.zip › Figure_6/Figure_6C/Slo_ladder.tif]

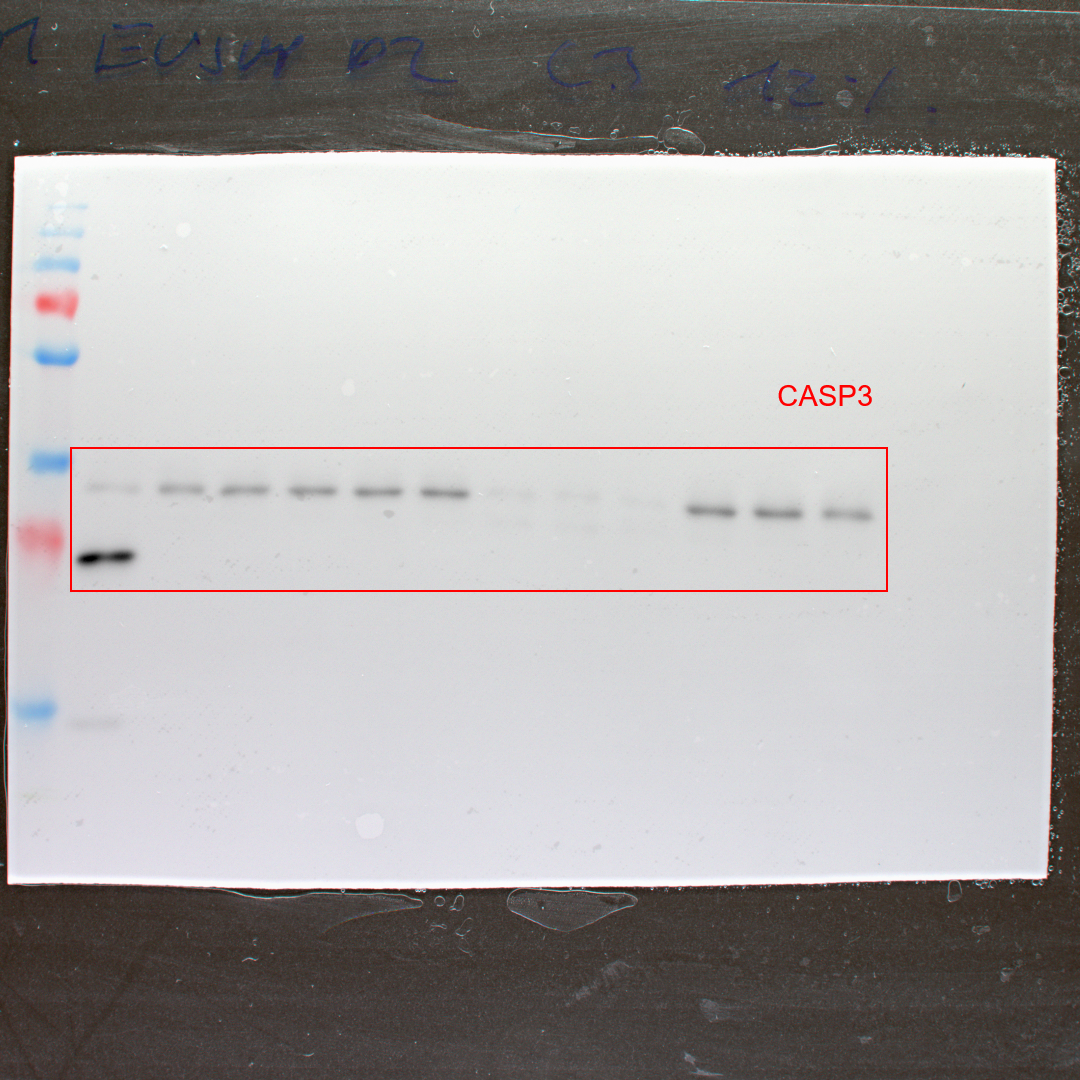

Supplement: Supplementary file 9 — Source data Fig. 6 [file 44319_2025_558_MOESM9_ESM.zip › Figure_6/Figure_6D/1_CASP3_ladder.tif]

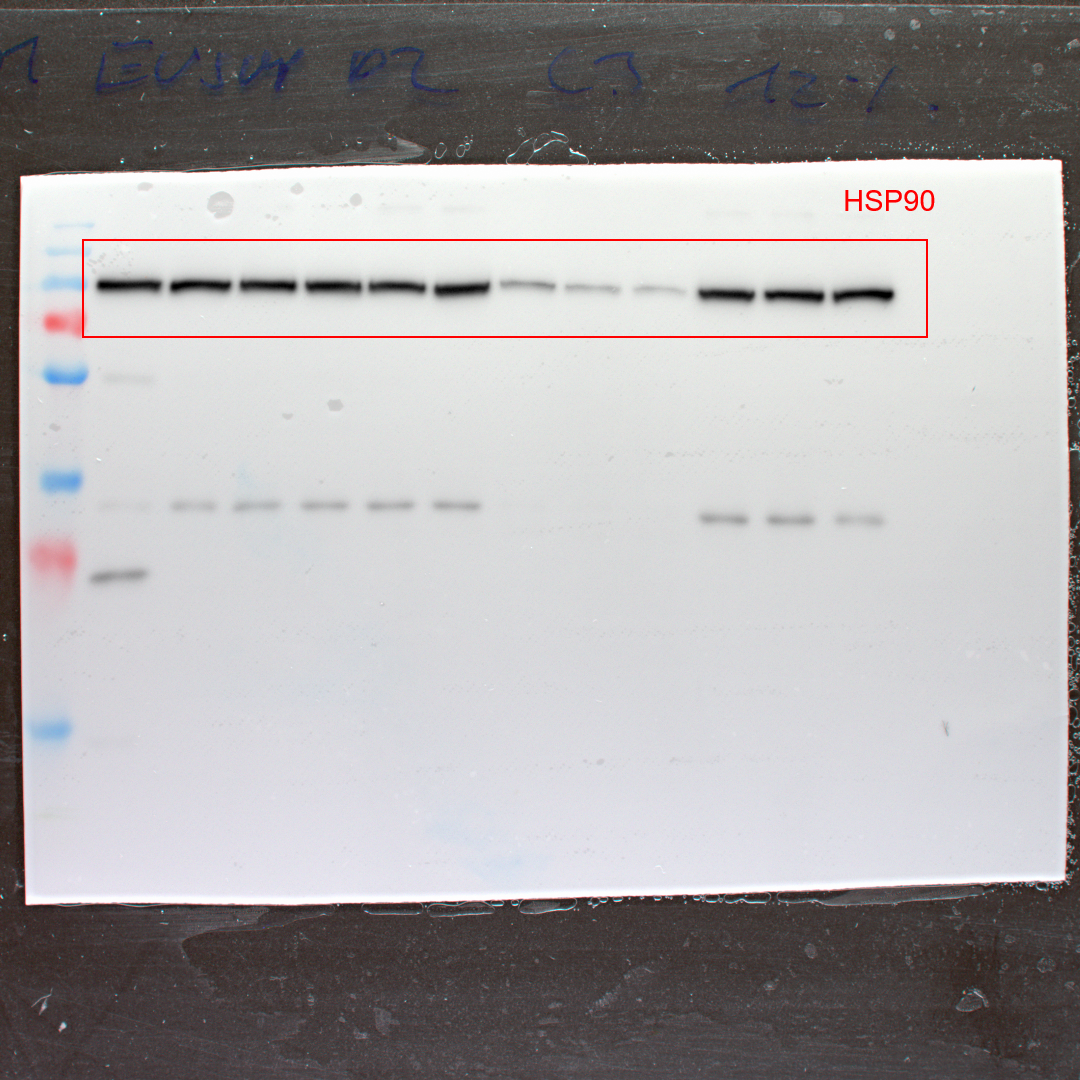

Supplement: Supplementary file 9 — Source data Fig. 6 [file 44319_2025_558_MOESM9_ESM.zip › Figure_6/Figure_6D/1_HSP90_ladder.tif]

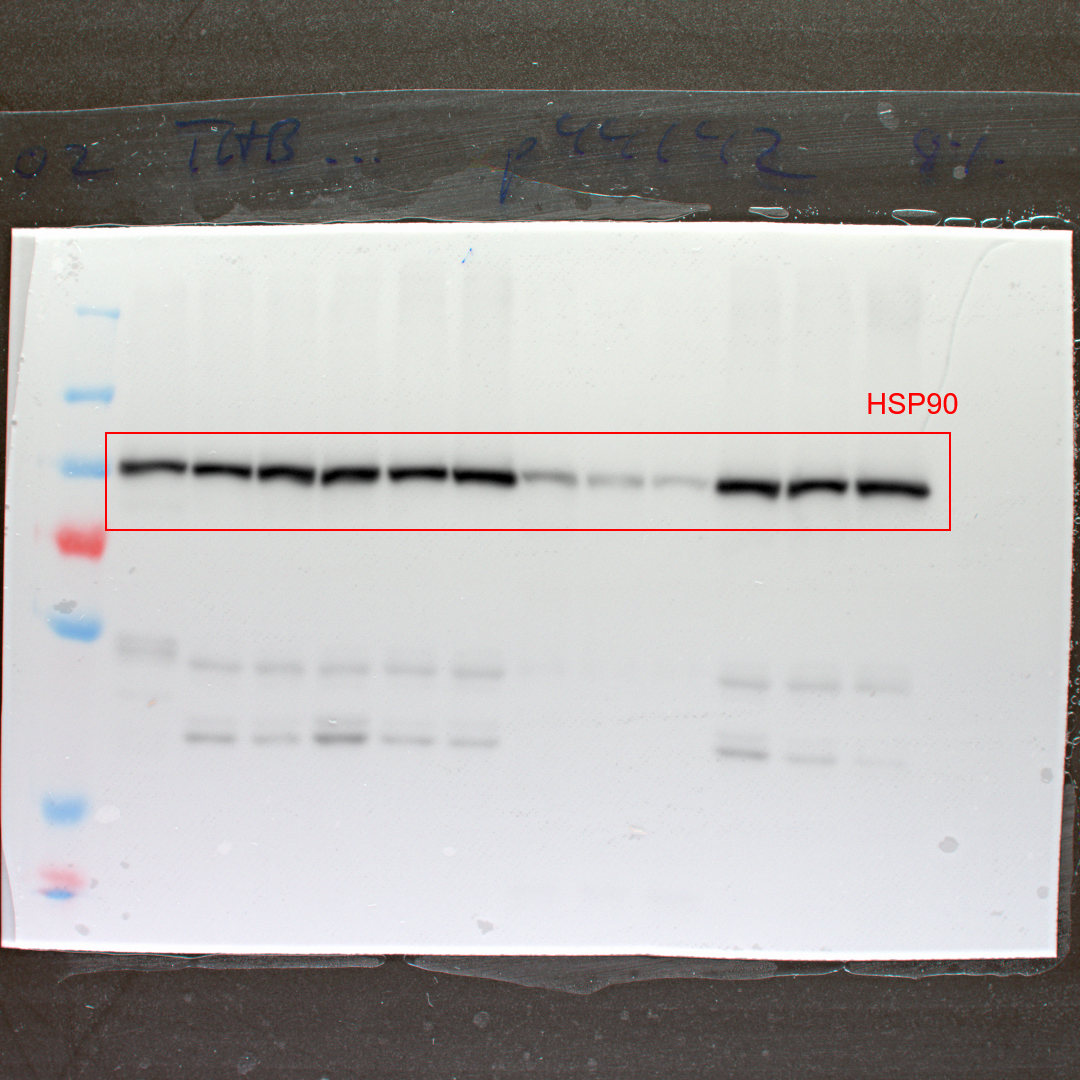

Supplement: Supplementary file 9 — Source data Fig. 6 [file 44319_2025_558_MOESM9_ESM.zip › Figure_6/Figure_6D/2_HSP90_ladder.tif]

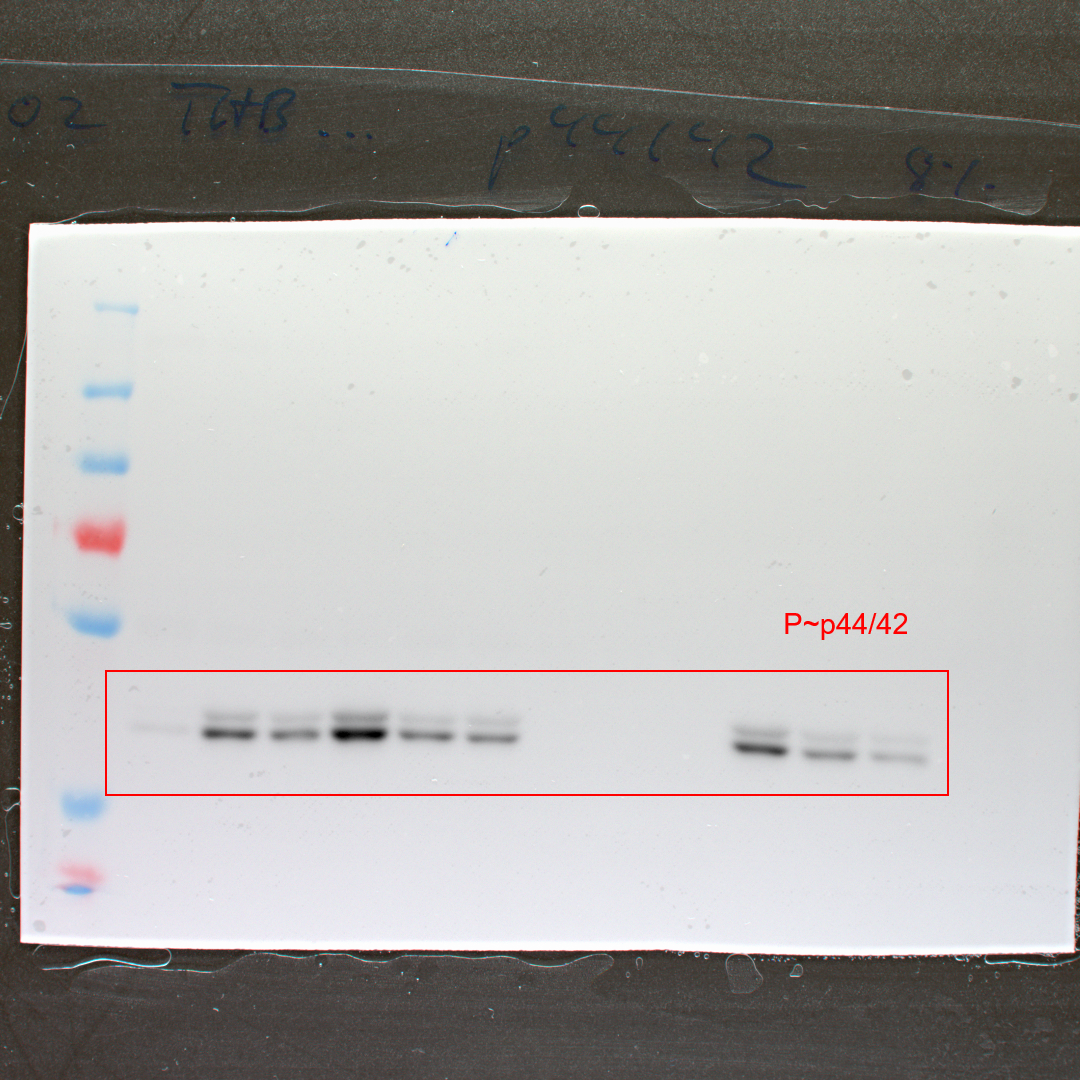

Supplement: Supplementary file 9 — Source data Fig. 6 [file 44319_2025_558_MOESM9_ESM.zip › Figure_6/Figure_6D/2_P_p44_42_ladder.tif]

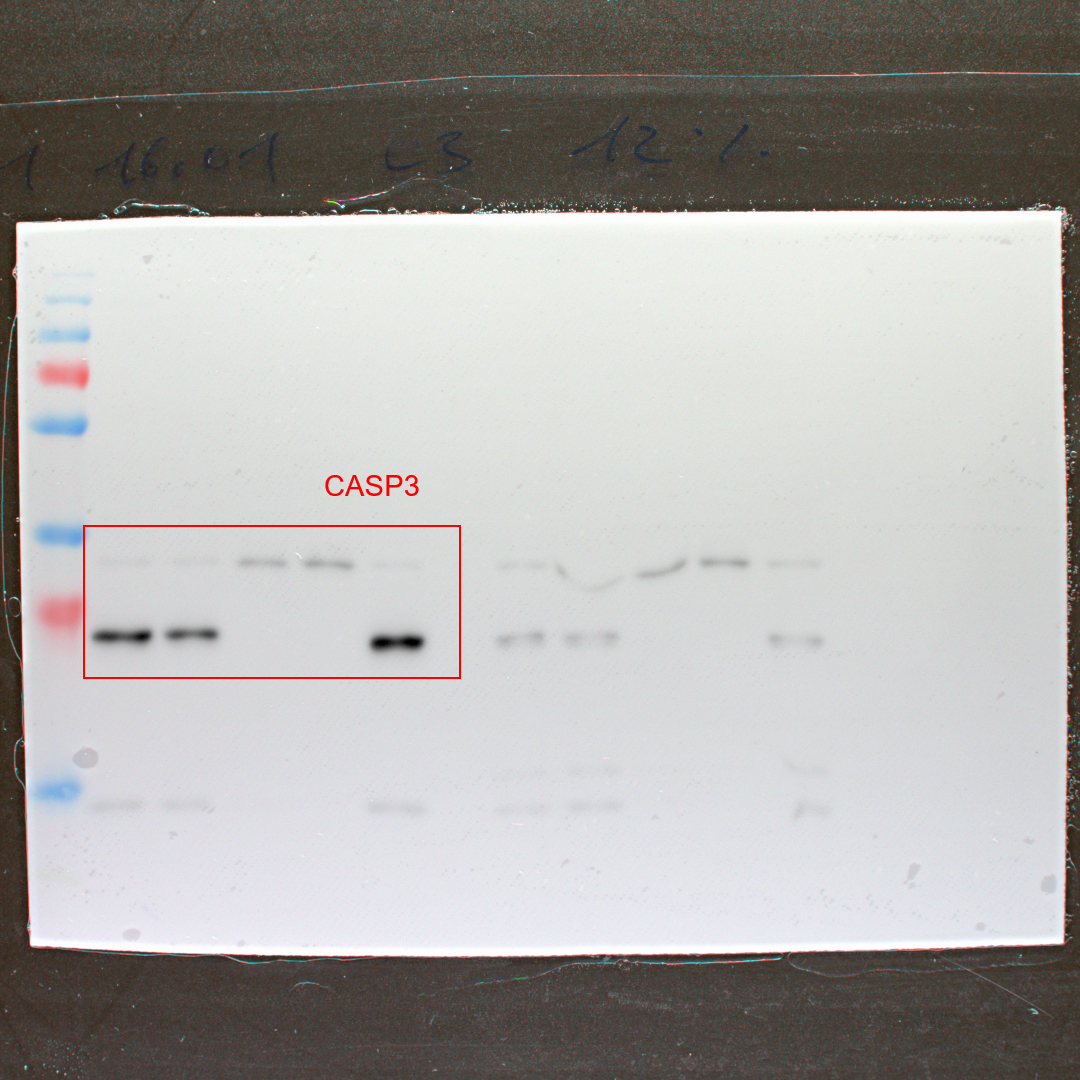

Supplement: Supplementary file 9 — Source data Fig. 6 [file 44319_2025_558_MOESM9_ESM.zip › Figure_6/Figure_6J/1_CASP3_ladder.tif]

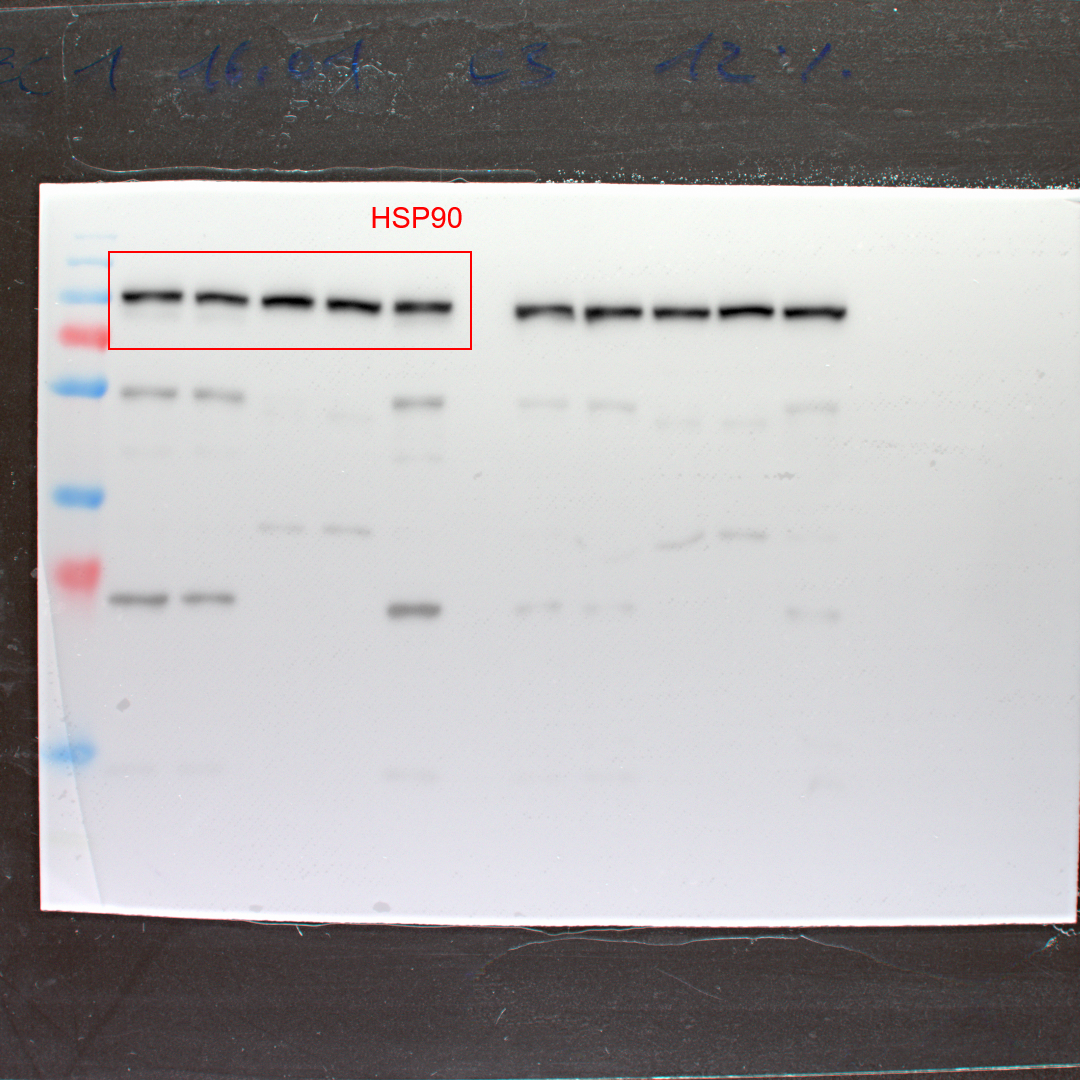

Supplement: Supplementary file 9 — Source data Fig. 6 [file 44319_2025_558_MOESM9_ESM.zip › Figure_6/Figure_6J/1_HSP90_ladder.tif]

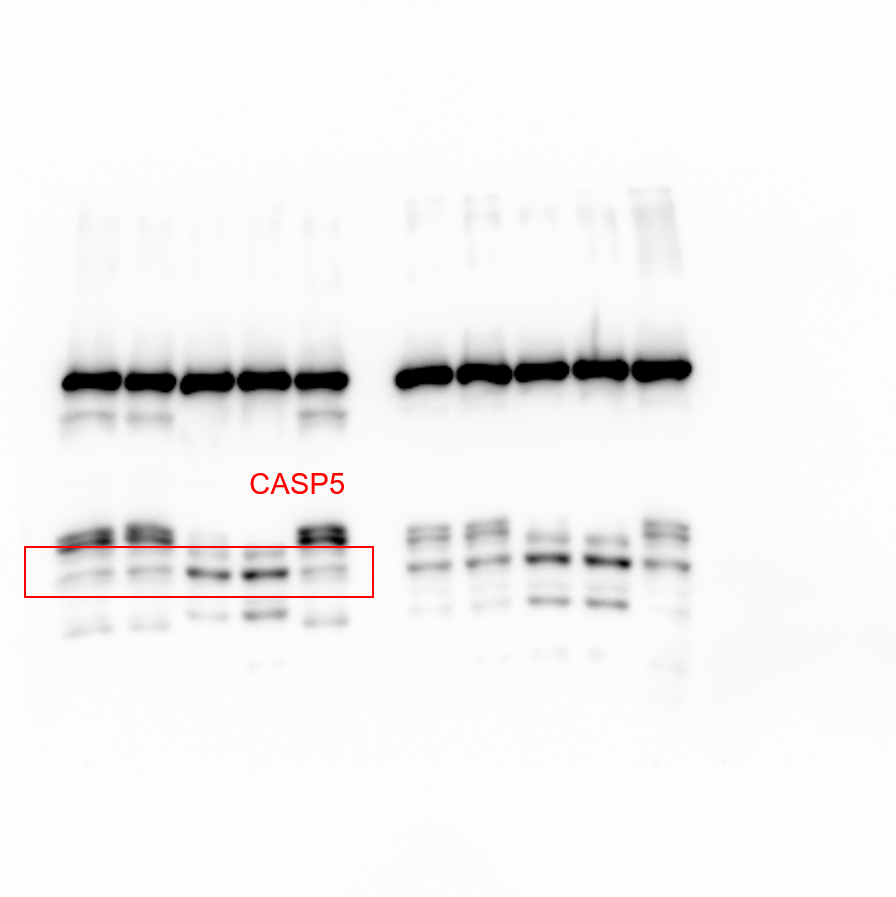

Supplement: Supplementary file 9 — Source data Fig. 6 [file 44319_2025_558_MOESM9_ESM.zip › Figure_6/Figure_6J/2_CASP5_longer_exposure.tif]

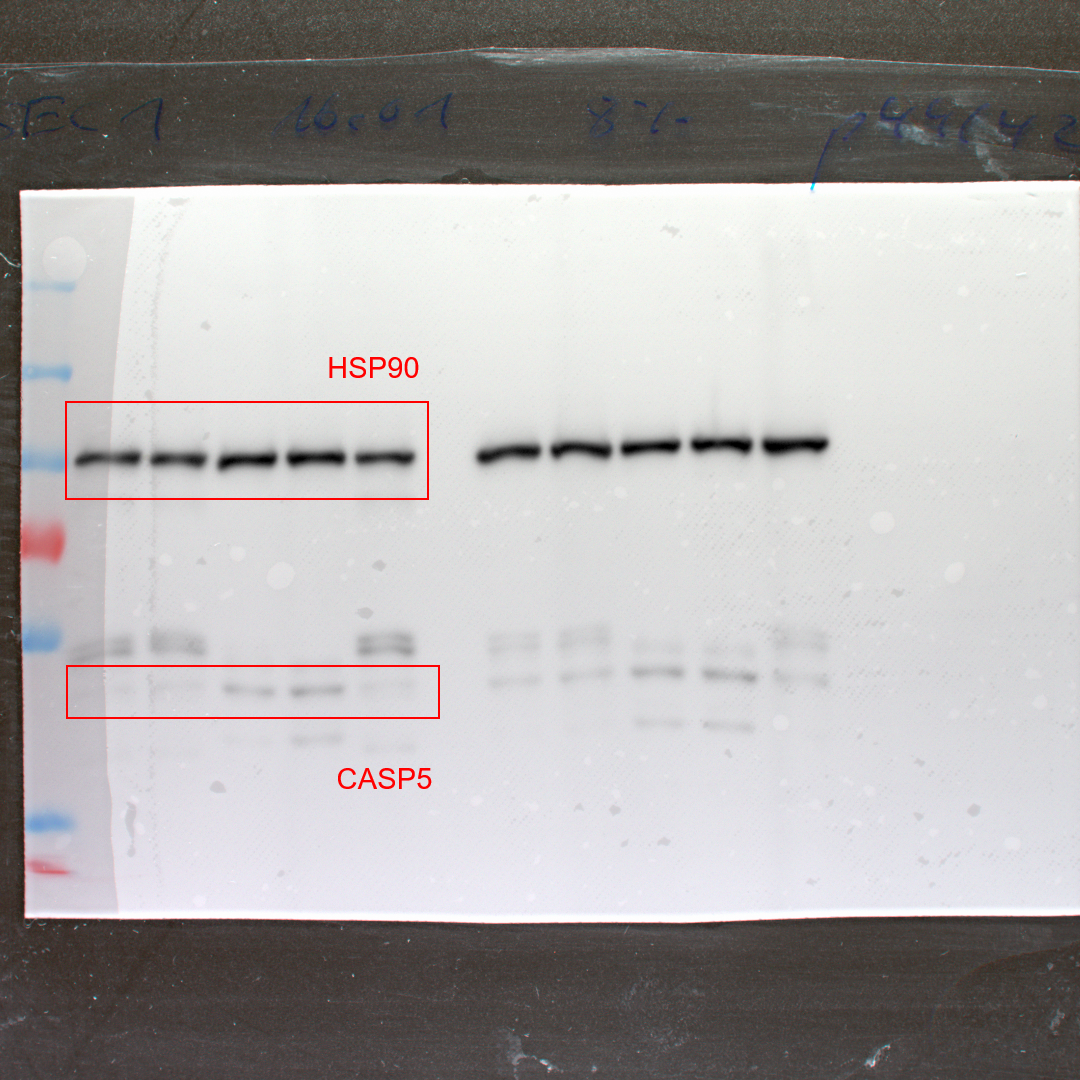

Supplement: Supplementary file 9 — Source data Fig. 6 [file 44319_2025_558_MOESM9_ESM.zip › Figure_6/Figure_6J/2_HSP90_CASP5_ladder.tif]

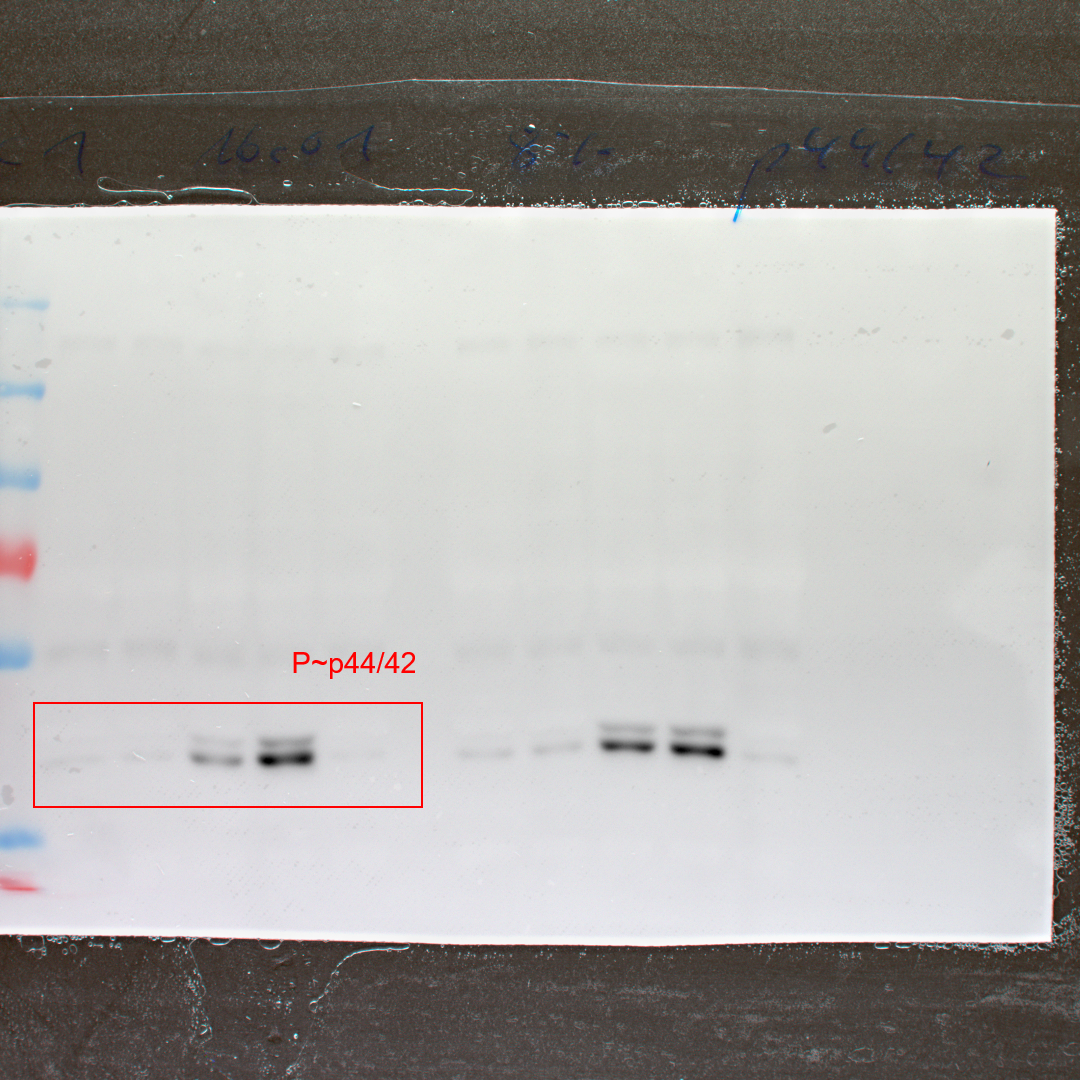

Supplement: Supplementary file 9 — Source data Fig. 6 [file 44319_2025_558_MOESM9_ESM.zip › Figure_6/Figure_6J/2_P-p44_42_ladder.tif]
