# Supplementary material for: Streptococcus pyogenes EVs induce the alternative inflammasome via caspase-4/-5 in human monocytes
Source: EMBO Rep. 2025 Sep 8;26(19):4847–85. doi: 10.1038/s44319-025-00558-7 (PMC12508482; doi:10.1038/s44319-025-00558-7)
Supplement: Supplementary file 10 — Expanded View Figures [file 44319_2025_558_MOESM10_ESM.pdf]

Expanded View Figures

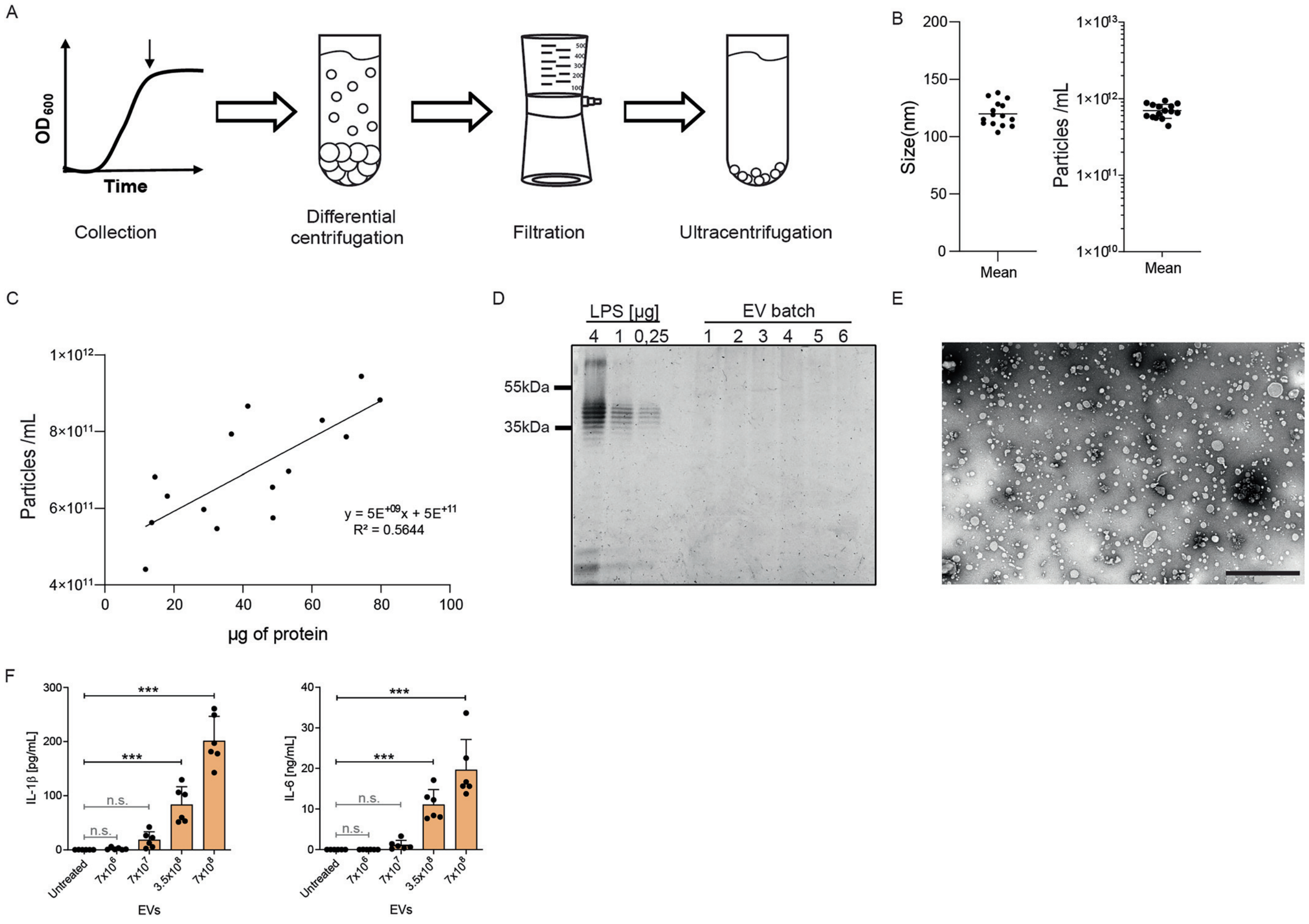

Figure EV1. Purification, quantification, and characterization of bacterial EVs used in this study.

(**A**) Schematic protocol of the purification strategy used for EVs. (**B**) Mean size (left) and concentration (right) of *Spy* EVs ($n = 15$) characterized by nanoparticle tracker analysis (NTA). (**C**) Linear correlation between particles/mL (measured by NTA) and the amount of protein of the same batches (Bradford assay, $n = 15$). (**D**) LPS analysis of EV preparations separated by SDS PAGE followed by Pro-Q Emerald 300 staining. Smooth LPS standard from *E. coli* serotype O55:B5 with characteristic ladder pattern was used as positive control. (**E**) *Spy* EV preparation imaged by transmission electron microscopy (TEM, scale bar: 1 μm). (**F**) IL-1β and IL-6 released by human monocytes stimulated with increasing amounts of *Spy* EVs for 18 h. Bars represent the mean ± SD of six biological replicates. $P < 0.0001$ (for all comparisons). Data information: (**F**) One-way ANOVA was applied with Holm–Šídák correction for multiple comparisons. ***$P \leq 0.001$, n.s. not significant.

© The Author(s)

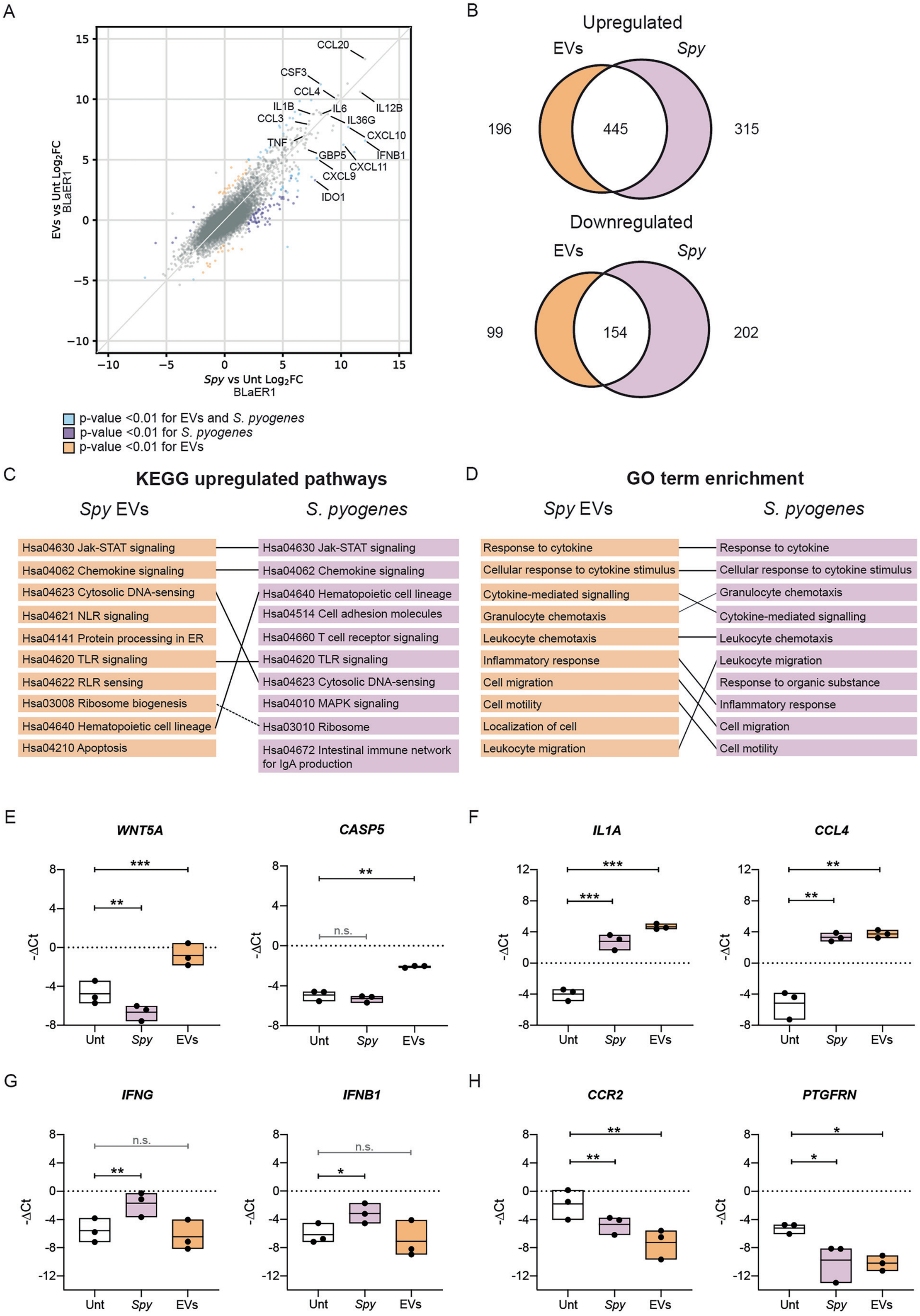
A
CCL20
CSF3
CCL4
IL1B
IL6
IL12B
CCL3
IL36G
CXCL10
TNF
IFNB1
GBP5
CXCL11
CXCL9
IDO1
EVs vs Unt Log2FC BLaER1
Spy vs Unt Log2FC BLaER1
p-value <0.01 for EVs and S. pyogenes
p-value <0.01 for S. pyogenes
p-value <0.01 for EVs
B
Upregulated
EVs
Spy
196
445
315
Downregulated
EVs
Spy
99
154
202
C
KEGG upregulated pathways
Spy EVs
S. pyogenes
Hsa04630 Jak-STAT signaling
Hsa04062 Chemokine signaling
Hsa04623 Cytosolic DNA-sensing
Hsa04621 NLR signaling
Hsa04141 Protein processing in ER
Hsa04620 TLR signaling
Hsa04622 RLR sensing
Hsa03008 Ribosome biogenesis
Hsa04640 Hematopoietic cell lineage
Hsa04210 Apoptosis
Hsa04630 Jak-STAT signaling
Hsa04062 Chemokine signaling
Hsa04640 Hematopoietic cell lineage
Hsa04514 Cell adhesion molecules
Hsa04660 T cell receptor signaling
Hsa04620 TLR signaling
Hsa04623 Cytosolic DNA-sensing
Hsa04010 MAPK signaling
Hsa03010 Ribosome
Hsa04672 Intestinal immune network for IgA production
D
GO term enrichment
Spy EVs
S. pyogenes
Response to cytokine
Cellular response to cytokine stimulus
Cytokine-mediated signalling
Granulocyte chemotaxis
Leukocyte chemotaxis
Inflammatory response
Cell migration
Cell motility
Localization of cell
Leukocyte migration
Response to cytokine
Cellular response to cytokine stimulus
Granulocyte chemotaxis
Cytokine-mediated signalling
Leukocyte chemotaxis
Leukocyte migration
Response to organic substance
Inflammatory response
Cell migration
Cell motility
E
WNT5A
CASP5
F
IL1A
CCL4
G
IFNG
IFNB1
H
CCR2
PTGFRN
-ΔCt
Unt
Spy
EVs
n.s.

 © The Author(s)

Figure EV2. RNA sequencing of monocytes: pathway analysis and RTqPCR of specific genes.

(**A**) Scatterplot comparison of Log_2 fold change (FC) (treatment vs untreated control, Unt) in transcript abundance for *S. pyogenes*-infected (x-axis) or EV-treated samples (y-axis) in BLaER1 cells (related to Fig. 1). Grey dots represent transcripts commonly regulated but with an FC < | 2 | . Blue dots denote commonly regulated transcripts with a FC ≥ | 2 | . Orange and purple dots indicate differentially expressed genes for either *Spy* EVs or *S. pyogenes*, respectively. (**B**) Venn diagrams of differentially transcribed genes in BLaER1 cells after stimulation with *Spy* EVs or *S. pyogenes* for 4 h. Numbers indicate the total amount of genes for each category. (**C**, **D**) Top ten upregulated KEGG pathways (**C**) or GO terms (**D**) are shown for *Spy* EV-treated versus untreated monocytes and *S. pyogenes*-treated versus untreated monocytes. Solid lines indicate same pathway and dashed lines indicate a related pathway. (**E–H**) Quantitative real time PCR of selected genes. Floating bar plots displaying the negative ΔCt values of three biological replicates. The central line represents the mean, the borders represent the minimum and the maximum values. A dotted line indicates the mean expression of the housekeeping genes (*GAPDH/TUBB*). (**E**) Genes upregulated by *Spy* EV treatment. (*WNT5A*) *P* = *0.0029, P* = *0.0004*. (*CASP5*) *P* = *0.0028*. (**F**) Genes upregulated by *S. pyogenes* and *Spy* EV treatments. (*IL1A*) *P* = *0.0003* (for both comparisons). (*CCL4*) *P* = *0.0014* (for both comparisons). (**G**) Genes upregulated by *S. pyogenes* treatment. (*IFNG*) *P* = *0.0025*. (*IFNB*) *P* = *0.0308*. (H) Genes downregulated for both *S. pyogenes* and *Spy* EV treatments. (*CCR2*) *P* = *0.0055* (*Spy*), *P* = *0.0010* (EVs). (*PTGRFN*) *P* = *0.0130* (for both comparisons). Data information: (**E–H**) One-way ANOVA with Holm–Šídák correction for multiple comparisons was applied for statistical analyses. *$P \leq 0.05$, **$P \leq 0.01$, ***$P \leq 0.001$, n.s. not significant.

© The Author(s)

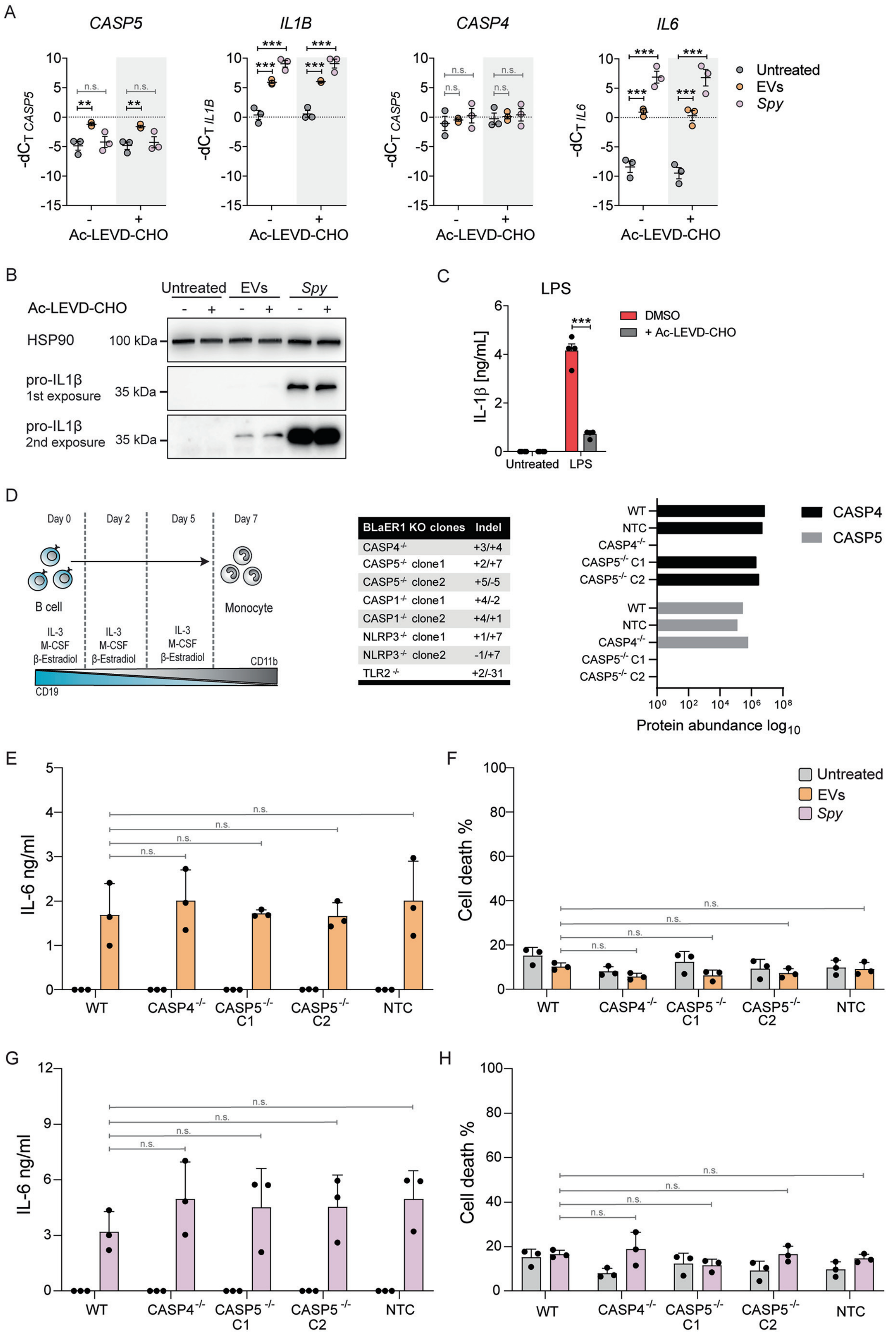

BLaER1 KO clones	Indel
CASP4-/-	+3/+4
CASP5-/- clone1	+2/+7
CASP5-/- clone2	+5/-5
CASP1-/- clone1	+4/-2
CASP1-/- clone2	+4/+1
NLRP3-/- clone1	+1/+7
NLRP3-/- clone2	-1/+7
TLR2-/-	+2/-31

 © The Author(s)

Figure EV3. BLaER1 knock-out characterization.

(**A**) Quantitative real time PCR of selected genes in human monocytes. Shown are mean ± SD of three biological replicates. A dotted line indicates the mean expression of the housekeeping genes (*GAPDH/TUBB*). (*CASP5*) $P = 0.0013$ (−), $P = 0.0018$ (+). (*IL1B*) $P = 0.0006$ (EVs for both comparisons), $P = 0.0001$ (*Spy*, for both comparisons). (*IL6*) $P = 0.0001$ (EVs for both comparisons), $P < 0.0001$ (*Spy*, for both comparisons). (**B**) Immunoblot analysis of pro-IL-1β in human monocytes. Cells were either left untreated or preincubated with the caspase-4/-5 inhibitor Ac-LEVD-CHO. Representative of three biological replicates. (**C**) IL-1β in response to LPS in supernatants from human monocytes either left untreated or preincubated with Ac-LEVD-CHO at 18 h. Shown are mean ± SD of three biological replicates. $P < 0.0001$ (**D**) Graphical representation of the differentiation protocol of BLaER1 cells as well as CRISPR-Cas9 mutagenesis of BLaER1 cells. The existence of frameshift mutations on each allele of the *CASP4*$^{-/-}$, *CASP5*$^{-/-}$, *TLR2*$^{-/-}$, *CASP1*$^{-/-}$, and *NLRP3*$^{-/-}$ clones was assessed using next generation sequencing. The guide RNAs used for genome editing are shown in the Reagents and Tools Table. Mass spectrometry analysis was used to confirm the absence of caspase-4 and caspase-5 from their respective KO clones compared to wild-type or non-targeted control (NTC) BLaER1 cells. (**E–H**) IL-6 and LDH released from BLaER1 WT, caspase-4 KO (*CASP4*$^{-/-}$), caspase-5 KO clones 1 and 2 (*CASP5*$^{-/-}$ C1 or C2), and non-targeted cells (NTC). BLaER1 cells were left untreated or were stimulated for 18 h with either *Spy* EVs or *S. pyogenes*. The percentage of LDH released from the positive control is shown as a measure of cell death. Bars represent the mean ± SD of three biological replicates. Data information: (**A**, **C**, **E–H**) Two-way ANOVA with Holm–Šídák correction for multiple comparisons was applied for statistical analyses. $^{**}P \le 0.01$, $^{***}P \le 0.001$, n.s. not significant.

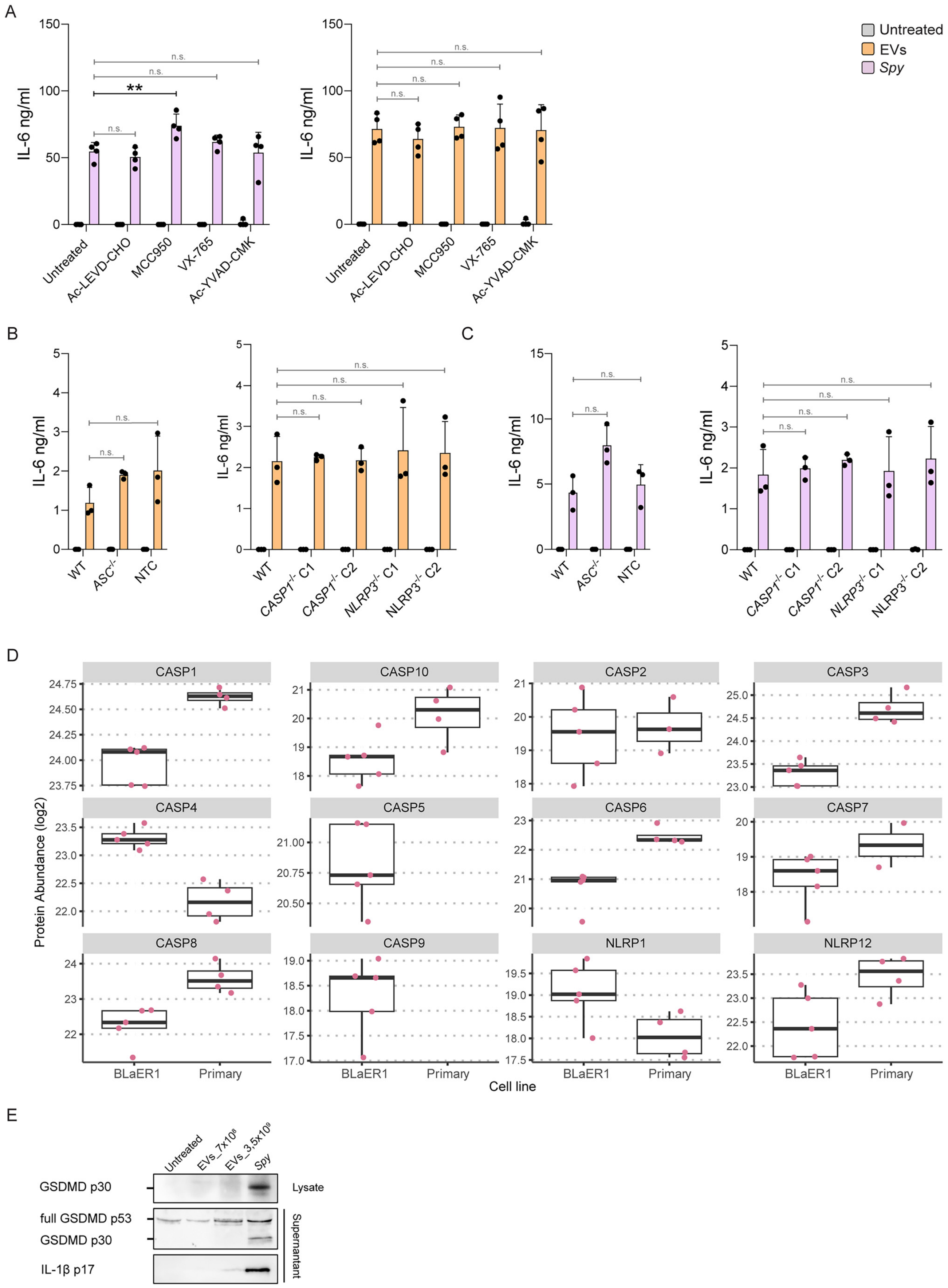
A
Untreated
EVs
Spy
IL-6 ng/ml
n.s.
**
Untreated
Ac-LEVD-CHO
MCC950
VX-765
Ac-YVAD-CMK
B
C
WT
ASC-/-
NTC
CASP1-/- C1
CASP1-/- C2
NLRP3-/- C1
NLRP3-/- C2
D
CASP1
CASP10
CASP2
CASP3
CASP4
CASP5
CASP6
CASP7
CASP8
CASP9
NLRP1
NLRP12
Protein Abundance (log2)
BLaER1
Primary
Cell line
E
Untreated
EVs_7x10^8
EVs_3.5x10^9
Spy
GSDMD p30
full GSDMD p53
GSDMD p30
IL-1β p17
Lysate
Supernatant

Figure EV4. Cytokine controls and cell death of monocytes and BLaER1 cells: canonical inflammasome.

(**A**) IL-6 in supernatants from human monocytes either left untreated or preincubated with Ac-LEVD-CHO, MCC950, VX-765, or Ac-YVAD-CMK. Cells were then left unstimulated or challenged with *S. pyogenes* or its EVs for 18 h. Bars represent the mean ± SD of four biological replicates. $P = 0.0011$. (**B**, **C**) IL-6 released from BLaER1 WT, PYCARD KO (*ASC*$^{-/-}$), NLRP3 KO clones 1 and 2 (*NLRP3*$^{-/-}$), Caspase-1 KO clones 1 and 2 (*CASP1*$^{-/-}$), and non-targeted control (NTC) cells stimulated with *Spy* EVs or infected with *S. pyogenes* for 18 h. Bars represent the mean ± SD of three biological replicates. (**D**) Box plot showing caspase and NLRP protein levels differentially expressed in BLaER1 cells (5 biological replicates) and primary human monocytes (4 biological replicates). Each dot represents the protein level of an individual sample. The boxplot displays the distribution of the data with the box representing the interquartile range (IQR) between the 25th (Q1) and 75th (Q3) percentiles. The line inside the box indicates the median (50th percentile). Whiskers extend to the smallest and largest values within 1.5 × IQR below Q1 and above Q3, respectively. Data points outside this range are shown individually as outliers. In total, 6472 proteins were quantified in at least 3 biological replicates of BLaER1 cells, and 6092 proteins were quantified in primary monocytes. (**E**) Immunoblots displaying GSDMD and IL-1β in human monocytes either left untreated or challenged with *Spy* EVs or *S. pyogenes* for 18 h. Representative of four biological replicates. Data information: (**A–C**) Two-way ANOVA with Holm–Šídák correction for multiple comparisons was applied for statistical analyses. $^{**}P \leq 0.01$, n.s. not significant.

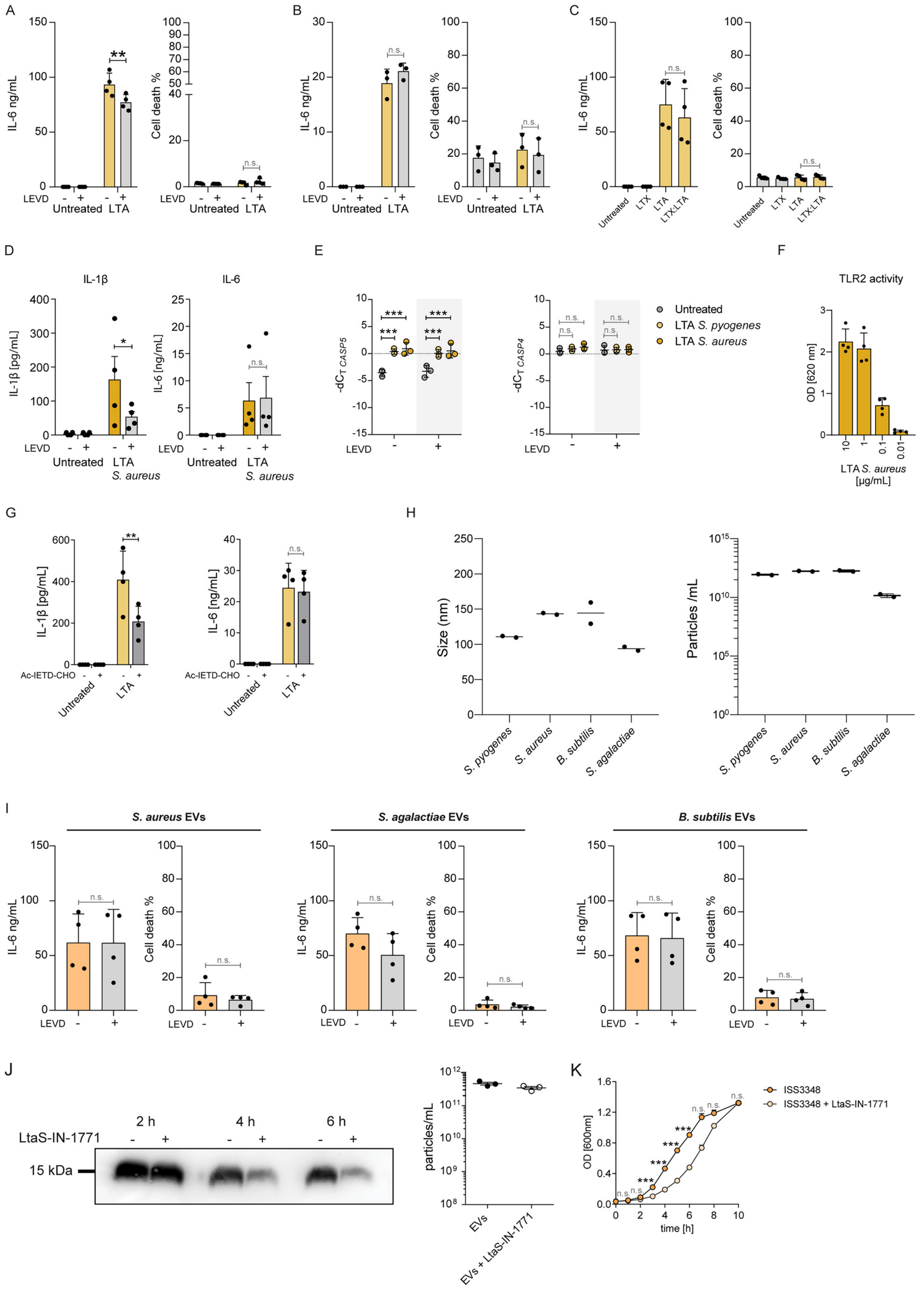
A
IL-6 ng/mL
Cell death %
LEVD - + - +
Untreated LTA
**
n.s.
B
IL-6 ng/mL
Cell death %
LEVD - + - +
Untreated LTA
n.s.
C
IL-6 ng/mL
Cell death %
Untreated
LTX
LTA
LTX:LTA
n.s.
D
IL-1β
IL-6
IL-1β [pg/mL]
IL-6 [ng/mL]
LEVD - + - +
Untreated LTA
S. aureus
*
n.s.
E
-dCT CASP5
-dCT CASP4
***
n.s.
LEVD - +
Untreated
LTA S. pyogenes
LTA S. aureus
F
TLR2 activity
OD [620 nm]
10
1
0.1
0.01
LTA S. aureus
[µg/mL]
G
IL-1β [pg/mL]
IL-6 [ng/mL]
Ac-IETD-CHO - + - +
Untreated
LTA
**
n.s.
H
Size (nm)
Particles /mL
S. pyogenes
S. aureus
B. subtilis
S. agalactiae
I
S. aureus EVs
S. agalactiae EVs
B. subtilis EVs
IL-6 ng/mL
Cell death %
LEVD - +
n.s.
J
2 h
4 h
6 h
LtaS-IN-1771
- + - + - +
15 kDa
particles/mL
EVs
EVs + LtaS-IN-1771
K
ISS3348
ISS3348 + LtaS-IN-1771
OD [600nm]
time [h]
***
n.s.

 © The Author(s)

Figure EV5. Cytokine controls and cell death of monocytes and BLaER1 cells: role of LTA in EV-dependent monocyte activation.

(**A**) IL-6 and LDH release at 18 h from monocytes that were left untreated or incubated with Ac-LEVD-CHO before the addition of *S. pyogenes* LTA. Shown are mean ± SD of four biological replicates. $P = 0.0069$. (**B**) IL-6 and LDH release at 18 h from BLaER1 cells that were left untreated or incubated with Ac-LEVD-CHO prior to addition of purified *S. pyogenes* LTA. Shown are mean ± SD of three biological replicates. (**C**) IL-6 and LDH release at 18 h from monocytes that were left untreated or treated with *S. pyogenes* LTA, either directly applied (LTA) or transfected using Lipofectamine LTX (LTx:LTA). Shown are mean ± SD of four biological replicates. (**D**) IL-1β and IL-6 release at 18 h from monocytes that were left untreated or incubated with Ac-LEVD-CHO before the addition of *S. aureus* LTA. Shown are mean ± SD of four biological replicates. $P = 0.0374$. (**E**) Quantitative real time PCR of *CASP5* and *CASP4*. Shown are mean ± SD of three biological replicates. A dotted line indicates the mean expression of the housekeeping genes (*GAPDH/TUBB*). $P < 0.0001$ (for all comparisons). (**F**) TLR2 activity in HEK-Blue TLR2 cells after stimulation with *S. aureus* LTA for 18 h. Baseline represents untreated cells. Bars represent the mean ± SD of four biological replicates. (**G**) IL-1β and IL-6 released by human monocytes that were either left untreated or preincubated with the caspase-8 inhibitor Ac-IETD-CHO. Cells were then left unstimulated or treated with *S. pyogenes* LTA for 18 h. Bars represent the mean ± SD of four biological replicates. $P = 0.0066$. (**H**) Mean size and particles/mL of *S. pyogenes*, *S. aureus, B. subtilis*, and *S. agalactiae* EVs ($n = 2$). (**I**) IL-6 and LDH release from monocytes either left untreated or preincubated with Ac-LEVD-CHO before the addition of EVs from *S. aureus*, *S. agalactiae*, or *B. subtilis* for 18 h. Shown are mean ± SD of four biological replicates. (**J**) Immunoblot analysis of LTA in bacterial pellets treated $-/+$ LtaS-IN-1771 and particles/mL of corresponding *S. pyogenes* EVs. Representative image of 2 biological replicates is shown. (**K**) *S. pyogenes* growth in the presence of LtaS-IN-1771 in THB media. Shown are mean ± SD of three biological replicates. (3 h) $P = 0.0010$, (4h-8h) $P < 0.0001$. Data information: (ABDEGK) Two-way ANOVA was applied with Holm–Šídák correction for multiple comparisons. (**C**) One-way ANOVA was applied with Holm–Šídák correction for multiple comparisons. (**I–H**) Statistical significance was assessed using paired *t* tests. *$P \le 0.05$, **$P \le 0.01$, ***$P \le 0.001$, n.s. not significant.

© The Author(s)
